# Ere, a Family of Short Interspersed Elements in the Genomes of Odd-Toed Ungulates (Perissodactyla)

**DOI:** 10.3390/ani14131982

**Published:** 2024-07-05

**Authors:** Ilia G. Ustyantsev, Sergey A. Kosushkin, Olga R. Borodulina, Nikita S. Vassetzky, Dmitri A. Kramerov

**Affiliations:** 1 Center for Precision Genome Editing and Genetic Technologies for Biomedicine, Engelhardt Institute of Molecular Biology, Russian Academy of Sciences, 119991 Moscow, Russia; 2 Laboratory of Eukaryotic Genome Evolution, Engelhardt Institute of Molecular Biology, Russian Academy of Sciences, 119991 Moscow, Russia

**Keywords:** SINE, retroposon, retrotransposon, RNA polymerase III, transcription terminator, polyadenylation, Equidae, Rhinocerotidae, Tapiridae, Perissodactyla

## Abstract

**Simple Summary:**

Short Interspersed Elements (SINEs) are small pieces of DNA that can move around in the genetic material of living things. They can modulate the genome function, e.g., induce hereditary diseases in humans and animals. In mammals, some SINEs have specific signals that help them make more copies of themselves. Scientists found a type of SINE called Ere in horses, rhinoceroses, and tapirs. They discovered that Ere SINEs have different versions with unique features. By studying these features, they could see how these SINEs have been active in the evolution of these animals. In particular, they found certain sequences in Ere SINEs that are important for making more copies of themselves.

**Abstract:**

Short Interspersed Elements (SINEs) are eukaryotic retrotransposons transcribed by RNA polymerase III (pol III). Many mammalian SINEs (T^+^ SINEs) contain a polyadenylation signal (AATAAA), a pol III transcription terminator, and an A-rich tail in their 3′-end. The RNAs of such SINEs have the capacity for AAUAAA-dependent polyadenylation, which is unique to pol III-generated transcripts. The structure, evolution, and polyadenylation of the Ere SINE of ungulates (horses, rhinos, and tapirs) were investigated in this study. A bioinformatics analysis revealed the presence of up to ~4 × 10^5^ Ere copies in representatives of all three families. These copies can be classified into two large subfamilies, EreA and EreB, the former distinguished by an additional 60 bp sequence. The 3′-end of numerous EreA and all EreB copies exhibit a 50 bp sequence designated as a terminal domain (TD). The Ere family can be further subdivided into subfamilies EreA_0TD, EreA_1TD, EreB_1TD, and EreB_2TD, depending on the presence and number of terminal domains (TDs). Only EreA_0TD copies can be assigned to T^+^ SINEs as they contain the AATAAA signal and the TCTTT transcription terminator. The analysis of young Ere copies identified by comparison with related perissodactyl genomes revealed that EreA_0TD and, to a much lesser extent, EreB_2TD have retained retrotranspositional activity in the recent evolution of equids and rhinoceroses. The targeted mutagenesis and transfection of HeLa cells were used to identify sequences in equine EreA_0TD that are critical for the polyadenylation of its pol III transcripts. In addition to AATAAA and the transcription terminator, two sites in the 3′ half of EreA, termed the β and τ signals, were found to be essential for this process. The evolution of Ere, with a particular focus on the emergence of T^+^ SINEs, as well as the polyadenylation signals are discussed in comparison with other T^+^ SINEs.

## 1. Introduction

Short Interspersed Elements (SINEs) or short retrotransposons are non-autonomous mobile genetic retroelements less than 600 bp in length that are transcribed by RNA polymerase III (pol III) [1,2,3]. SINEs are characteristic of the vast majority of multicellular organisms. A genome may contain one or more SINE families; the number of SINE copies of a given family may reach one million. SINE copies that make up a family share a common evolutionary origin and show significant nucleotide sequence similarity. The copies of SINE subfamilies share even greater sequence similarity. Subfamilies arise from sequence changes in SINEs that favor their amplification in the genome. New copies of SINEs in genomes are produced by reverse transcription (retrotransposition) carried out by an enzyme encoded in Long Interspersed Elements (LINEs) present in the same genome. Most families of SINEs originated from tRNAs; accordingly, the 5′-terminal (head) portions of such SINEs resemble the nucleotide sequences of a particular tRNA species. Both tRNA genes and tRNA-derived SINEs are transcribed by pol III from an internal promoter. Such pol III promoters consist of 11 bp boxes A and B separated by 30–40 bp. The 3′-terminal part of SINEs is important for the recognition of their transcripts by reverse transcriptase encoded by LINEs. In placental mammals, most SINE families use L1 reverse transcriptase for their retrotransposition, which requires a poly(A) tail at the end of the SINE.

SINEs may play an important role in the evolution of genes and genomes. They are distributed throughout the genome, including genes. Many copies of SINEs can be found in introns in both orientations; they are less common in the 3′-untranslated regions of exons. Accordingly, SINE sequences can be co-transcribed by RNA polymerase II as part of pre-mRNA molecules. The integration of new SINE copies into genes, including introns and regulatory regions, can sometimes disrupt gene function [2,4,5]. However, these insertions often are selectively neutral or sometimes even positive and later become fixed in a population. This fixation might be due to beneficial effects on the gene transcription [6,7,8], splicing [2,9,10], and polyadenylation [11,12] of pre-mRNAs. Noncoding antisense RNAs carrying SINE sequences can enhance the translation of mRNAs transcribed from the same genomic loci [13,14]. SINE transcripts synthesized by pol III appear to play an important role in helping cells cope with stressors [15,16].

While studying SINE families from mammalian genomes, we noticed that some of them are characterized by AATAAA signals and pol III transcription terminators (TCT_≥3_ or T_≥4_) upstream of the poly(A) tail [17]. These T residues in AATAAA and in the transcription terminators underlie the name to the T^+^ class of such SINEs, whereas SINEs lacking these two signals were assigned to the T^−^ class. All twelve families of SINEs assigned to the T^+^ class are found only in placental genomes [17,18,19]. We analyzed eight families of T^+^ SINEs and showed that their transcripts synthesized by pol III can be polyadenylated via an AAUAAA-dependent pathway [20,21]. Previously, only RNA polymerase II transcripts, namely mRNAs and many protein-noncoding RNAs, were thought to undergo such polyadenylation. (The mechanisms of mRNA 3′-end processing and subsequent polyadenylation are now very well understood [22,23,24,25]). Our experiments with B2, Dip, and Ves SINEs (from the mouse, jerboa, and bat genomes, respectively) showed that in addition to the AATAAA sequence (polyadenylation signal, PAS), two other sequences contribute to poly(A) synthesis at the 3′-ends of these SINE transcripts [21]. The first (β signal) is located just downstream of the B box of the pol III promoter, and the second (τ signal) is located upstream of the sequence containing AATAAA signals. In B2 transcripts, the τ signal represents the binding site of the polyadenylation factor CFIm [19]. In Dip and Ves, the role of τ signals is played by polypyrimidine motifs [21]; similar polypyrimidine motifs are characteristic of four other families of T^+^ SINEs. The β and τ signals contribute approximately equally to the polyadenylation efficiency and function independently of SINE sequences other than AATAAA [21,26]. The long poly(A) tail (A_>20_) in SINE transcripts is a prerequisite for retrotransposition, which is performed by the L1-encoded reverse transcriptase [27,28,29,30]. In the case of SINE families (e.g., Alu in primates), which we categorize as T^−^, the reverse transcription of SINE RNA initiates at the poly(A) tail transcribed from genomic DNA rather than generated by polyadenylation [2,31,32]; such a mechanism can be termed T^−^ retrotransposition. We believe that in T^+^ SINEs, reverse transcription is primed at the poly(A) tail synthesized by the polyadenylation of the SINE transcript; we refer to this mechanism as T^+^ retrotransposition [5,20,33].

This work analyzes the SINE family Ere specific to odd-toed ungulates. Our interest in this SINE family stems from its assignment to the T^+^ class [17,21], based on the presence of a PAS and a transcription terminator in the initially described five Ere copies [34]. Later, Ere SINEs from the horse genome were divided into two families—EreA and EreB [18]. Nevertheless, Ere remains a poorly and fragmentarily studied SINE family [35,36]; therefore, we performed an extensive bioinformatics analysis of this SINE in the genomes of equids, rhinoceroses, and tapirs. It turned out that one of the EreA subfamilies belongs to the T^+^ class, while the other EreA subfamilies, as well as EreB, belong to the T^−^ class. The Ere SINEs have a number of interesting features and a complex structural organization. The experimental part of this work allowed us to identify the nucleotide sequences of the β and τ signals in the EreA SINEs from the horse genome.

## 2. Materials and Methods

### 2.1. Bioinformatics Methods

The following assemblies were downloaded from NCBI Genomes (https://www.ncbi.nlm.nih.gov/genome, accessed 10 January 2024): horse (*Equus caballus*), EquCab3.0; Przewalski horse (*Equus przewalskii*), Burgud; donkey (*Equus asinus asinus*), ASM303372v1; plains zebra (*Equus quagga*) isolate Etosha38, UCLA_HA_Equagga_1.0; northern white rhino (*Ceratotherium simum cottoni*), ASM2144216v1; black rhino (*Diceros bicornis minor*), mDicBic1.mat.cur; lowland tapir (*Tapirus terrestris*), TapTer_v1_BIUU; and Malayan tapir (*Tapirus indicus*), TapInd_v1_BIUU. Multiple sequence alignments were obtained using MAFFT [37] and edited in GeneDoc (http://www.nrbsc.org/gfx/genedoc/index.html, accessed 10 January 2024). We used custom scripts based on the Smith–Waterman algorithm implemented in the ssearch36 program from the FASTA package [38] to search for all genomic copies of SINEs with at least 65% identity and 90% length overlap with the query sequence.

SINE subfamilies were identified manually and/or using the custom script SubFam described elsewhere [33]. Average similarity was determined for 100 randomly selected sequences using the esl-alistat program (http://hmmer.org, accessed 10 January 2024).

SINE presence/absence loci in genomes were determined by mapping ~200 bp flanking regions of each SINE-containing locus using BWA-MEM [39]; matches were analyzed using various tools including SeqKit [40] and BEDtools [41]. The presence or absence of SINEs was determined by locus size and manually checked for representative loci.

RNA secondary structures were predicted using the RNAfold program of the RNAWebSuite (http://rna.tbi.univie.ac.at/cgi-bin/RNAWebSuite/RNAfold.cgi, accessed 10 January 2024).

### 2.2. Plasmid Constructs

Ere-T and Ere-C constructs (Figure S1) containing a native and inactivated PAS, respectively, were obtained and described previously [21]. Deletions and nucleotide substitutions were introduced into the Ere-T plasmid using the Phusion Site-Directed Mutagenesis Kit (Thermo Fisher Scientific, Waltham, MA, USA) according to the manufacturer’s protocol. The Sor/Ere-T plasmid (Figure S1) was constructed using the following procedures. (i) PCR of Sor SINE (Sar 4SA clone [17]); the reverse primer contained the *Bgl* II site. (ii) PCR amplification of the 78 bp Ere-T tail fragment; the direct primer contained the *Bgl* II site. (iii) Two amplified fragments were ligated at the *Bgl* II site and cloned into pGEM-T (Promega, Madison, WI, USA). Deletions were introduced into the Sor/Ere-T plasmid using the Phusion Site-Directed Mutagenesis Kit. The plasmids designed for transfection were isolated using the Plasmid Midi Kit (Qiagen, Hilden, Germany) according to the manufacturer’s protocol.

### 2.3. Cell Transfection and Northern Blot Analysis

HeLa cells (ATCC, CCL-2) were grown to an 80%-confluent monolayer in 60 mm Petri dishes using DMEM with 10% fetal bovine serum. Cells on each dish were transfected with 4 μg DNA of Ere-T or the same construct containing deletions and/or substitutions mixed with 10 μL of TurboFect reagent (Thermo Fisher Scientific, Waltham, MA, USA) according to the manufacturer’s protocol. Cells were transfected with plasmid Sor/Ere-T and derived constructs using the same protocol. Transfections were performed in triplicate. Cellular RNA was isolated 20 h after transfection using the guanidine thiocyanate method [42] and further purified by RNase-free DNase I treatment [20]. RNA samples (10 μg) obtained from each transfection were separated by electrophoresis in 6% polyacrylamide gel with 7 M urea, transferred to a nylon membrane (GVS, Bologna, Italy) by semi-dry electroblotting, and hybridized with an Ere or Sor probe labeled with α[^32^P]dATP, Taq-polymerase, and reverse primers [21]. Northern hybridization conditions were described elsewhere [21]. Hybridization signals were quantified by scanning the membranes in a phosphorimager (Image Analyzer Typhoon FLA 9000; GE Healthcare Bio-sciences, Uppsala, Sweden).

## 3. Results

### 3.1. Structure and Subfamilies of Ere

**Figure 1:**
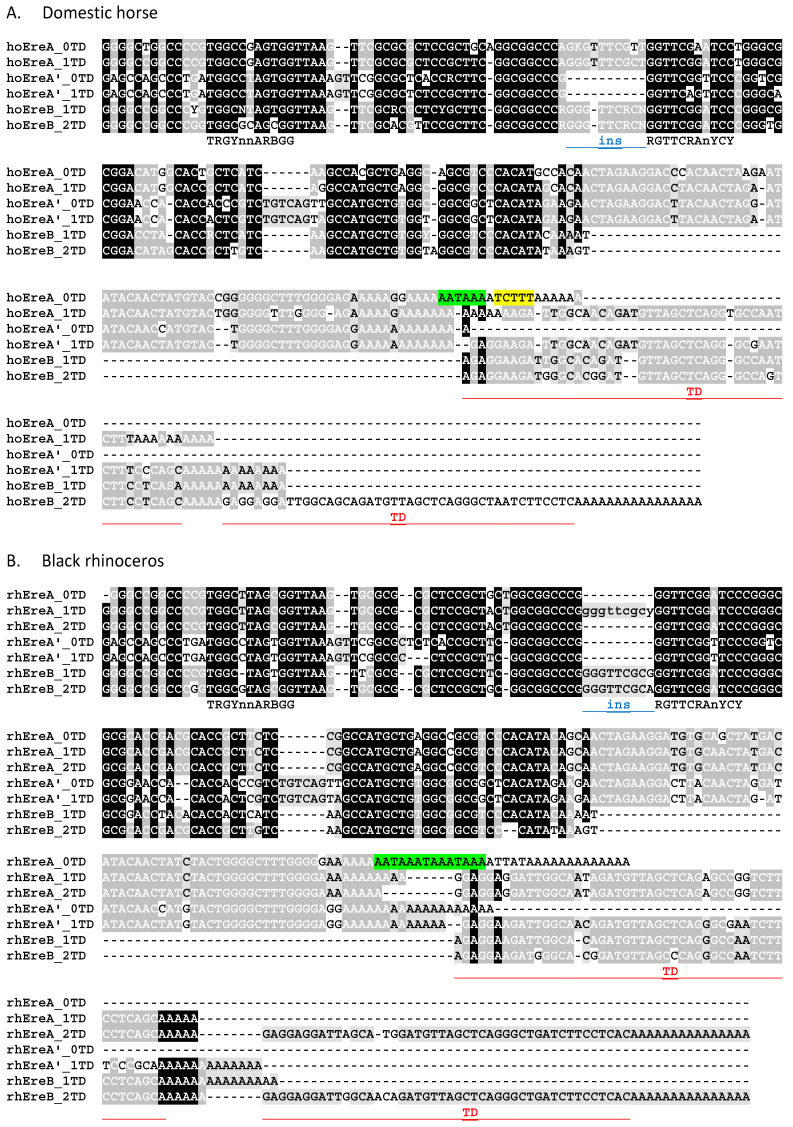

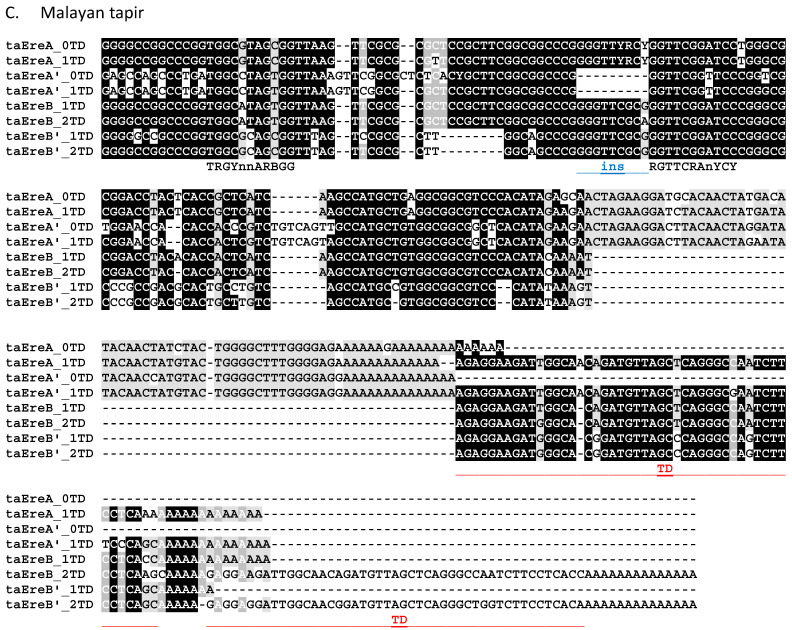
The alignments of the consensus sequences of Ere subfamilies from the genomes representing three subfamilies of odd-toed ungulates (Perissodactyla). (**A**) Domestic horse, (**B**) black rhinoceros, and (**C**) Malayan tapir. The consensus boxes A and B of the pol III promoter are shown below the alignment. The 9 bp insertion (ins) is marked with a blue line. The PAS and terminator are highlighted in green and yellow, respectively. The TD, which stands for terminal domain, is underlined in red.

**Figure 2:**
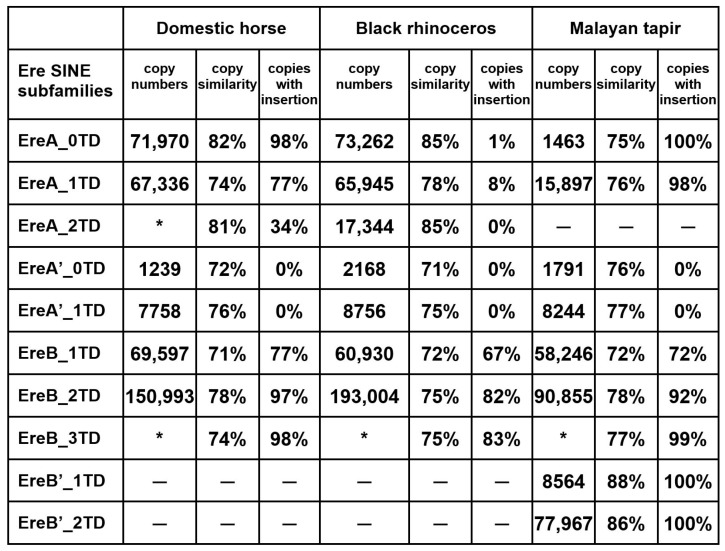
The number of copies of Ere subfamilies, their average similarity, and the percentage of copies with the 9 bp insertion. Subfamilies with fewer than 1000 genomic copies are marked with an asterisk.

An exhaustive search for copies of the SINEs EreA and EreB was performed in the genomes of all three families (Equidae, Rhinocerotidae, and Tapiridae) of the order Perissodactyla. The total number of these SINEs was determined to be 372,250 in the genome of the domestic horse (*Equus caballus*), 35,1904 in the Przewalski horse (*E. przewalskii*), 332,379 in the domestic donkey (*E. asinus asinus*), 372,381 in the plains zebra (*E. quagga*), 420,814 in the black rhino (*Diceros bicornis*), 394,631 in the white rhino (*Ceratotherium simum*), 264,893 in the Malayan tapir (*Tapirus indicus*), and 245,363 in the lowland tapir (*T. terrestris*). The copies of these SINEs were divided into subfamilies, and while we previously considered EreA and EreB to be two distinct SINEs [18], in this work, we conclude that they belong to two related, albeit highly divergent, subfamilies of the Ere SINE. Alignments of the consensus sequences of Ere subfamilies from the domestic horse, black rhinoceros, and Malayan tapir genomes were performed. Three of these alignments, each consisting of 6–8 Ere subfamily consensus sequences, are shown in Figure 1. Figure 2 summarizes the copy number of each subfamily and the average sequence similarity within each subfamily, indicating the relative age of the subfamilies studied.

The consensus sequence analysis of the Ere subfamilies revealed the following. (1) EreA and EreB are alignable; they are similar in many respects, but the 3′ half of EreA contains a ~60 bp sequence that is absent in EreB. (2) The head of all Ere subfamilies is 73–78% similar to the Glu(CTC) tRNA sequence from which Ere likely evolved (Figure S2). (3) Several Ere subfamilies (four in horse, two in rhinoceros, and six in tapir) are characterized by the presence of a 9–10 bp insertion upstream of the promoter B box in most copies of these subfamilies (Figure 1 and Figure 2); sequence similarity suggests that this insertion arose by the tandem duplication of the B box. (4) The 3′ region in most Ere subfamilies contains a specific ~50 bp sequence that we have termed the terminal domain (TD). Our division of Ere into subfamilies was largely based on the presence and number of TDs. The number of TDs can vary, and the suffix to the subfamily name reflects this, e.g., 0TD, 1TD, 2TD, and rarely 3TD (Figure 1).

All EreB contains terminal domains; most contain two TDs, and such EreB_2TDs are the most abundant in the odd-toed ungulate genomes examined (Figure 2). EreA either has no TDs or contains a single one (only rhinoceros has a significant number of EreA_2TDs). TDs and the rest of Ere are separated by oligo(A) linkers (Figure 1). In tapir genomes, apart from the usual EreA and EreB, Ere subfamilies with specific substitutions, deletions, or insertions in the SINE head were detected; they were named EreA’ and EreB’ (Figure 1). While EreA’ was also detected in the horse and rhinoceros genomes, EreB’ was restricted to the tapir genome, where it was even more abundant than EreB (Figure 2). 

It is worth explaining why we identified Ere subfamilies in the horse genome rather than using consensus sequences from Repbase. This database contains 14 records of Ere sequences, some of which are highly similar and cannot be reliably distinguished. A 9–10 bp insertion upstream of the promoter B box is a variable feature (Figure 1 and Figure 2) and should not be used as a universal subfamily marker. Our subfamilies are discernible by long sequences (TD and 60 bp EreA-specific region) and several stable nucleotide substitutions and indels. For reference, our subfamilies (in bold) have the following closest counterparts: **EreA_0TD**, ERE1; **EreA_1TD**, ERE1B/ERE1C; **EreA’_0TD**, ERE4; **EreA’_1TD**, ERE4B; **EreB_1TD**, ERE3C2/ERE3C3/ERE3C4/ERE3D; and **EreB_2TD**, ERE2/ERE3/ERE3B/ERE3B2/ERE3C.

**Figure 3:**
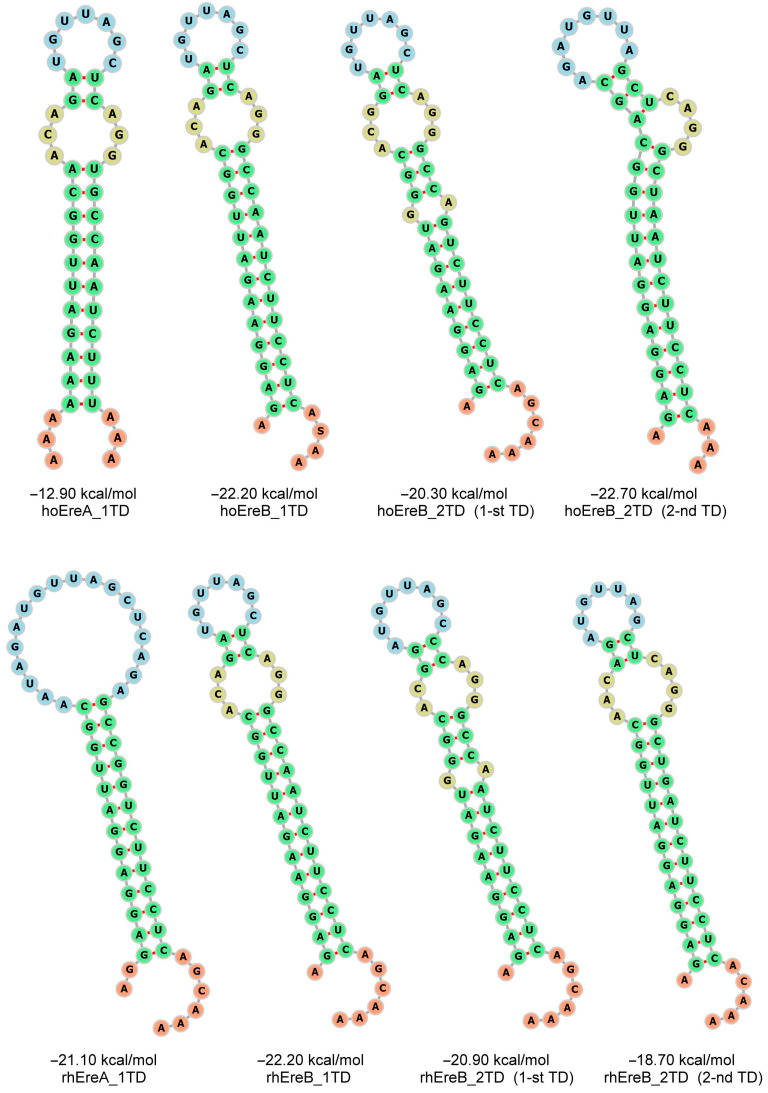
Predicted secondary structure of terminal domain (TD) of Ere SINE transcripts from domestic horse and black rhinoceros. Consensus sequences depicted in Figure 1A,B were folded. Their free energy values and corresponding Ere subfamilies are indicated below structures. TD structures of horse (ho) and rhinoceros (rh) are shown above and below, respectively. Blue indicates loop nucleotides, while green and yellow highlight paired and unpaired stem nucleotides, respectively.

The analysis of the consensus sequences of TDs revealed that their right and left halves are complementary to each other. In other words, TD sequences should form hairpin structures with a double helix length of up to 13 bp in the transcripts. The secondary structures of TDs from the Ere subfamilies of three ungulate species are shown in Figure 3 (horse and rhinoceros) and Figure S3 (tapir). The highly conserved secondary structure of TDs indicates the importance of this site, most likely for Ere retrotransposition.

### 3.2. Ere Subfamilies of T^+^ SINEs

Of all the Ere subfamilies discussed above, only EreA_0TD from the horses and rhinos, but not tapirs, possessed the features of T^+^ SINEs: they contained PASs (one in horse and three in rhinoceros) and the pol III transcription terminator (Figure 1). In horses, the EreA_0TD consensus has a moderately efficient terminator TCTTT (highlighted in yellow), whereas in rhinos, the consensus terminator is replaced by TTAT, which is clearly an inefficient terminator. However, as will be shown below (see Section 3.3), the analysis of the young rhino EreA_0TD copies revealed efficient terminators in them.

**Figure 4:**
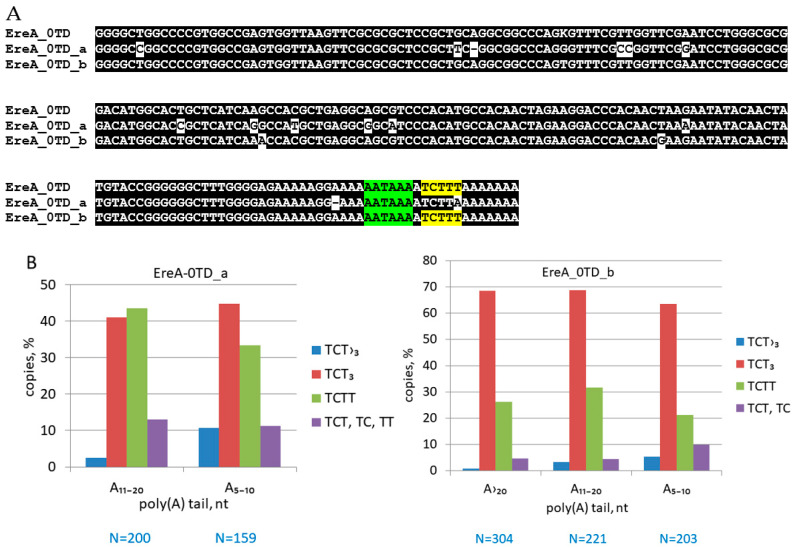
Two variants of equine SINE subfamily EreA_0TD. (**A**) Consensus sequences of EreA_0TD_a and EreA_0TD_b with PASs highlighted in green and terminator in yellow. (**B**) Distribution of pol III terminators (TCT_≥3_) or their rudiments among copies of EreA_0TD_a and EreA_0TD_b with poly(A) tails of different lengths. Number of copies analyzed is indicated at bottom as (N = number).

Horse EreA_0TD can be divided into two variants (a and b) with differing consensus sequences at 16 positions (Figure 4A). The average similarity to consensus is 80 and 86% for EreA_0TD_a and EreA_0TD_b, respectively, indicating a later emergence and reproduction of variant b compared to a. EreA_0TD_b copies account for 46% of all EreA_0TDs. We analyzed transcription terminators in EreA_0TD copies with A-tails of varying lengths (A_>20_, A_11–20_, and A_5–10_), similar to our approach with other T^+^ SINEs [5,33]. Young SINE copies are known to have long poly(A) tails, but the length of these tails decreases over time. Therefore, the length of A-tails can indicate the age of SINE copies [2,27,28,33]. Figure 4B shows that older EreA_0TD copies of both variants with short tails (A_5–10_) have a five times higher incidence of longer and more efficient terminators (TCT_>3_). However, this effect was more pronounced in other T^+^ SINEs (B2, Dip, Ves, and Can) that we analyzed similarly [5,33]. Up to 40% of copies of these SINEs with A_5–10_ tails had highly efficient terminators (TCT_>3_, TAT_>3_, or T_>4_) compared to only 5–10% of such copies in EreA_0TD (Figure 4B). We previously suggested that there may be a rarely employed mechanism to add T residues to T^+^ SINE terminators, which eventually make them longer and more efficient in many SINE copies over extended time periods [5,33]. However, it remains unclear why this process is less pronounced in EreA_0TD SINEs.

### 3.3. An Assessment of the Activity of Ere Subfamilies

To determine which Ere subfamilies were retrotranspositionally active in the recent evolutionary period, a bioinformatic search was conducted for Ere copies present in the genome of one odd-toed ungulate species and absent in the orthologous loci of another. These polymorphic SINE copies are relatively young since they integrated into the genome after the divergence of the two species. Table 1 summarizes the number of polymorphic copies of EreA and EreB identified by comparing the genomes of the four equid species. As expected, the lowest number of polymorphic Ere (1500 and 2445) was detected for the domestic horse *E. caballus* and Przewalski horse *E. przewalskii*, which diverged 45,000 years ago [43,44,45]. When comparing the genomes of these horses to those of the donkey *E. asinus* or the plains zebra *E. quagga* (which diverged from the horse branch 4.5 million years ago [45]), 5–10 times more copies were found. Additionally, the number of polymorphic copies of EreA was 10–20 times higher than that of EreB. It is worth noting that EreA_0TD copies accounted for the majority (at least 97%) of all polymorphic EreA. Polymorphic copies of EreB_2TD were approximately 10 times more frequent than those of EreB_1TD. Therefore, the EreA_0TD subfamily, specifically its “b” variant, is the most retrotranspositionally active, followed by EreB_2TD in the equid genomes.

A similar search for polymorphic Ere copies was conducted in the genomes of black and white rhinos (*D. bicornis* and *C. simum*) that diverged 6.8 million years ago [46,47] and in the genomes of two distant tapir species (*T. indicus* and *T. terrestris*) that diverged 22 million years ago [48,49] (Table 2). Unexpectedly, the abundance of polymorphic copies in the two rhinos showed a significant difference between Ere subfamilies. In black rhinoceros, EreB_2TD (57%) and EreA_0TD (39%) were active after species divergence, while in white rhinoceros, EreA_0TD dominated (87%) over EreB_2TD (11%). EreA_1TD and EreA_2TD showed little activity and together accounted for only 2–4% of all polymorphic Ere copies in these rhinos. The Malayan tapir appeared to have 2.4 times more polymorphic Ere copies than the lowland tapir, and all of these copies belonged to the EreB’_2TD subfamily. Thus, after the divergence of these two tapir species, only this subfamily retained retrotranspositional activity.

**Figure 5:**
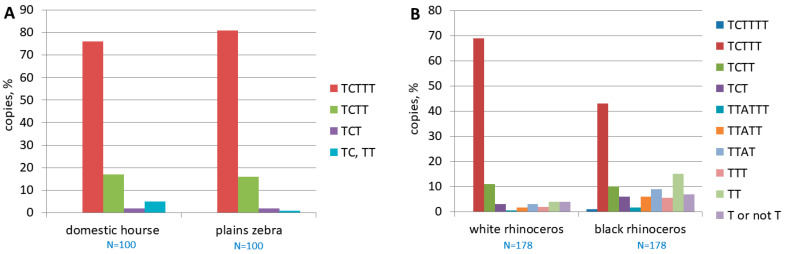
Distribution of pol III-terminators or their rudiments in samples of polymorphic EreA_0TD copies identified for genomes of domestic horse and plains zebra (**A**) or white and black rhinoceroses (**B**). Number of copies analyzed is indicated as N = number.

The analysis of the pol III transcription terminators of the polymorphic copies of EreA_0TD from the genomes of the domestic horse and plains zebra, as well as two rhinoceros species, showed the following (Figure 5). In horse, zebra, and white rhinoceros, copies with a full-length albeit moderately efficient TCTTT terminator predominated (70–80%). In black rhinoceros, this terminator was also predominant but far less abundant (43%). A rudimentary terminator, TCTT (10–17% copies), was found in all species; but in rhinoceroses, especially black rhinoceros (15% of copies), TTAT and TTATT sequences were often located in place of the terminator. Such sequences cannot function as transcription terminators and are probably rudiments of the TTATTT terminator, which also occurred in small numbers in the samples analyzed (Figure 5B).

EreA_0TD copies with the TCTTT terminator must be propagated by the T^+^ mechanism that involves transcription termination at TCTTT and subsequent RNA polyadenylation. Given the 50% efficiency of termination at this signal, it cannot be excluded that EreA_0TD copies can sometimes amplify by the T^−^ mechanism.

### 3.4. An Analysis of Young EreA_0TD Copies in the Horse Genome

After identifying EreA_0TD copies in the domestic horse genome that are absent in the orthologous loci of Przewalski horse, we tried to reveal the most similar copies among these young copies. The copies that differed from their common consensus in no more than two positions (the variable tail part was excluded from consideration) were considered similar and thus related to each other. Thirty-two groups (tribes) of such related copies of EreA_0TD were identified. Most of the tribes consisted of a small number of copies (less than 10), but a few included several tens of copies. The largest, and therefore active, tribe contained 111 copies. An exhaustive search for EreA_0TD copies (not only polymorphic ones) similar to the consensus of this largest tribe in the domestic horse genome revealed a total of 471 copies, i.e., about a quarter of these copies integrated into the genome after the divergence of *E. caballus* and *E. przewalskii*. Let us examine the structure of the tail sequences of young copies of EreA_0TD using the example of this most successful tribe, designated as #1.

Figure S4 shows the nucleotide sequences of all 111 copies of EreA_0TD of tribe #1, which are present in the genome of the domestic horse but absent in the orthologous loci of the Przewalskii horse. Almost all copies are flanked by perfect direct repeats representing target site duplication (TSD), consistent with the young age (<45,000 years) of these copies. The analysis of closely related copies of EreA_0TD allows us to evaluate the variation in their 3′-terminal sequences. A total of 6 out of 111 copies have lost their PAS through the substitution of T for a different nucleotide. In 12 copies, the PAS is slightly shifted due to the amplification of the A residues around it (this shift should not significantly affect PAS function). Two copies demonstrated a complete loss of the terminator, apparently by deletion. More than 75% of the copies have the TCTTT transcription terminator, and 20% of the copies carry a rudimentary terminator TCTT (Figures S4 and S5), which arises by terminator shortening occurring sometimes after pol III transcription and the subsequent formation of a daughter copy of the SINE [5,33]. Terminator rudiments such as TCT and TC, occasionally found in copies of EreA_0TD from tribe #1 (Figures S4 and S5), likely have a similar origin. The lengths of the poly(A) tails ranged from 10 to 40 nt. These data show the degree of variation in the 3′-terminal sequences in closely related copies of EreA_0TD that originated less than 45,000 years ago.

### 3.5. Identification of β and τ Signals in EreA_0TD

**Figure 6:**
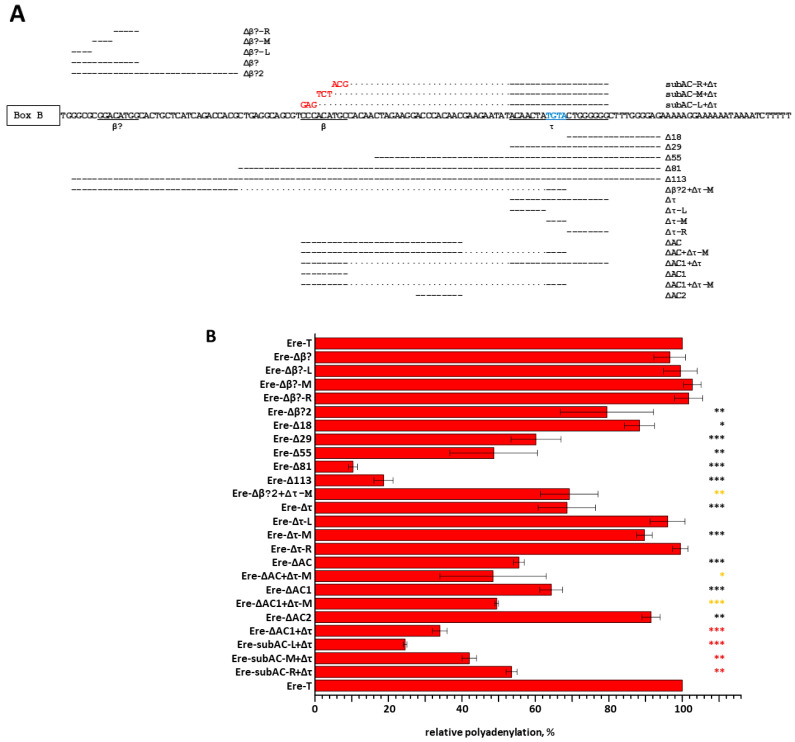
The effect of deletions and substitutions in putative β and τ regions on the polyadenylation of SINE Ere transcripts. The diagram in panel (**A**) displays the introduced deletions and substitutions. The middle part of the diagram shows the portion of the Ere nucleotide sequence (Ere-T) located upstream of box B. The putative β and τ regions are underlined (the TGTA site is highlighted in blue within the τ region). In the constructs, deleted nucleotides are marked by dashes above or below the sequence, and unaltered nucleotides between two stretches of altered (deleted or substituted) nucleotides are shown by dots. Substitutions are indicated in red above the wild-type sequence. The construct names are given on the right side. Panel (**B**) displays the relative polyadenylation level of the transcripts of Ere-based constructs with substitutions and deletions. The polyadenylation level of Ere-T is taken as 100% and is shown at the top and bottom of the diagram. The left side of the diagram indicates the names of the constructs. (Error bars, SD, n = 3). *** *p* ≤ 0.001; ** *p* ≤ 0.01; * *p* ≤ 0.05. The *p*-value was determined by a *t*-test. Black, brown, and orange asterisks indicate *p*-values determined for constructs that are derivatives of the Ere-T, Ere-Δτ, and Ere-Δτ-M constructs, respectively.

The nucleotide sequences (β and τ signals) within EreA_0TD that are required for the efficient polyadenylation of transcripts were identified experimentally for one copy of EreA_0TD in the horse genome. The plasmid carrying this copy of Ere (Figure S1) [21] was used to generate 24 constructs containing deletions and/or substitutions in the Ere sequence (Figure 6A). Additionally, a hybrid Sor/Ere SINE was created by combining a Sor sequence (a T^−^ SINE previously discovered from the shrew genome [17,21]) with the 78 bp 3′-terminal portion of Ere (Figure S1). Furthermore, nine different deletions were made to this Ere portion (Figure S6). HeLa cells were transfected with all resulting constructs. The isolated cellular RNA was analyzed by Northern hybridization using radioactively labeled probes complementary to Ere or Sor RNA (in the case of Sor/Ere constructs). Polyadenylated transcripts were revealed by Northern hybridization as heterogeneous RNA longer than the primary transcripts of the Ere constructs (Figures S6C and S7). Figure 6B displays the impact of deletions and substitutions on the polyadenylation efficiency of transcripts in comparison to the original SINE EreA_0TD (Ere-T construct).

A range of constructs (Ere-Δ18, -Δ29, -Δ55, -Δ81, and -Δ113) with deletions starting at 19 bp from the terminator and extending different distances upstream revealed that the sequences essential for the polyadenylation of Ere transcripts are located within the 81 bp deletion. This finding is also consistent with the following. The β signal in Ere is assumed to be located almost immediately downstream of the B box of the promoter by analogy with B2, Dip, and Ves. The site, labeled as ‘β?’ in Figure 6A, contains six nucleotides (ACATGG) that are part of the B2 β signal. However, deletions in the Ere-Δβ?, -Δβ?-L, -Δβ?-M, and -Δβ?-R regions did not reduce transcript polyadenylation. This suggests that the β signal is not located in this Ere region.

Next, we focused on the τ signal in Ere (Figure 6A). This was based on the site position, which is similar to that of the τ signal in B2; additionally, it contains TGTA, an RNA binding site for CFIm, similar to the B2 τ signal [19]. The Ere-Δ29 construct showed reduced polyadenylation (60% of control) and indicated the approximate left border of the τ signal. A 19 bp deletion in the proper τ signal (Ere-Δτ construct) resulted in polyadenylation that was 75% of the control. Surprisingly, a separate deletion of the three parts constituting the τ signal (Δτ-L, Δτ-M, Δτ-R) had almost no effect on polyadenylation. This suggests that the τ signal is tolerant to partial deletions. These findings were confirmed in experiments with the Sor/Ere construct and its derivatives carrying deletions in the Ere sequence (Figure S6). In this case, the effects of deletions were more significant because the 78-nucleotide sequence of Ere lacks another signal (see below) that contributes to the polyadenylation of transcripts of the original Ere. It is notable that the aforementioned TGTA is situated in the center of the τ signal, with its right portion comprising six G residues (Figure 6A).

Figure 6 shows that the Δ81 deletion resulted in a further decline in the polyadenylation of the Ere transcript (10%) compared to the Δ55 deletion (50%). This suggests that a sequence in the larger deletion includes another polyadenylation signal. Indeed, it contains a CCCACACATGC sequence resembling the β signal in B2 (ACCACATGG). The deletion of this AC-rich sequence (Ere-ΔAC1 construct) lowered polyadenylation to 65% of the control, indicating its significance. The deletion of this sequence together with the τ signal (Ere-ΔAC1+Δτ) reduced polyadenylation to 35%. (When studying the β signal, we often deleted the τ signal to maximize the effects of deletions and/or substitutions in the putative β signal.) Trinucleotide substitutions in this 9 bp sequence strongly affected polyadenylation. The substitution of CCC (Ere-subAC1-L+Δτ construct) had a particularly strong effect. Thus, the AC1 sequence is anticipated to represent a β signal (underlined in Figure 6A).

**Figure 7:**
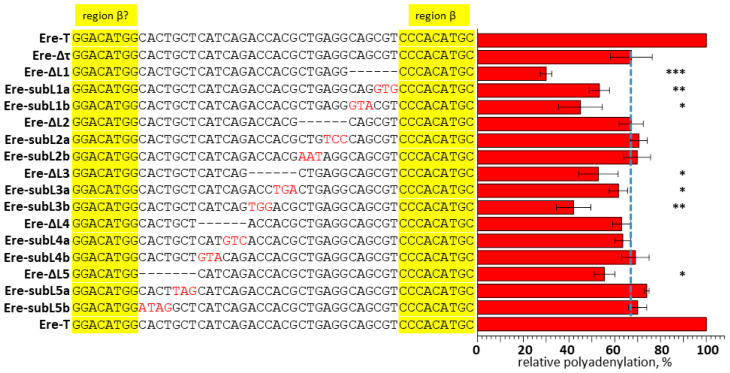
The effect of the Ere sequence upstream of the β region on polyadenylation. The sequence between the β? and β regions (highlighted in yellow) was subjected to 6 nt deletions or 3 nt substitutions. Hyphens indicate deleted nucleotides, and substituted nucleotides are shown in red. All deletions and substitutions were made in the construct with the deleted τ signal (Ere-Δτ); the names of the constructs used to transfect HeLa cells are indicated on the left. The red bars on the right indicate the relative polyadenylation of transcripts; the polyadenylation of the Ere-T transcript is taken as 100%. The blue dashed line marks the polyadenylation level of the Ere-Δτ transcript; the polyadenylation of the other RNA constructs should be compared with this, since all deletions and substitutions were introduced into Ere-Δτ. (Error bars, SD, *n* = 3.). *** *p* ≤ 0.001; ** *p* ≤ 0.01; * *p* ≤ 0.05. The *p*-value was determined by a *t*-test.

The sequences downstream of the AC1 site do not appear to play a significant role in polyadenylation (as demonstrated by, e.g., the Ere-ΔAC2 construct). Surprisingly, the region upstream of AC1 was found to contribute significantly to polyadenylation. This left (L) region was divided into five hexanucleotides (L1, L2, L3, L4, and L5), and each was deleted from the Ere-Δτ construct, resulting in constructs Ere-ΔL1 to Ere-ΔL5 (Figure 7). Trinucleotide substitutions (two variants) were introduced into each of these sites resulting in constructs labeled as Ere-subL1a, Ere-subL1b, Ere-subL2a, Ere-subL2b, and so on (Figure 7). The experiments demonstrated that the L1 hexanucleotide adjacent to AC1 contributed as much as the AC1 sequence to the polyadenylation efficiency (Figure 7 and Figure S8). In contrast, the L2 site appears to be insignificant for polyadenylation. However, the deletion or substitution of the L3 hexanucleotide (but not the next two, L4 and L5) also affected the polyadenylation efficiency (Figure 7). Hence, we found a bipartite β signal in EreA_0TD that is noticeably longer (6 + 15 bp) than the β signals (9 bp) in B2 and Ves SINEs. Furthermore, the β signal in Ere is located farther away from box B of the pol III promoter than in the SINEs we previously investigated.

## 4. Discussion

**Figure 8:**
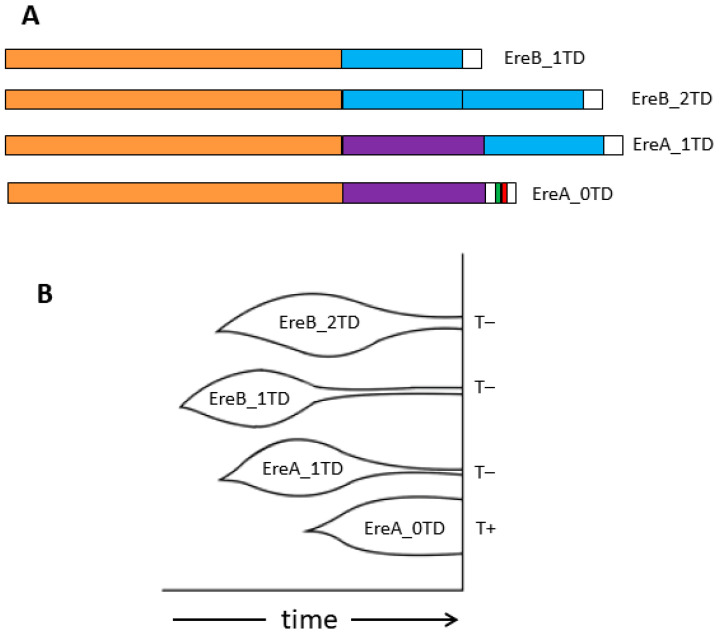
(**A**) Schematic structure of Ere SINE subfamilies. Similar nucleotide sequences are shown by equally colored rectangles: sequences common to all Ere subfamilies, orange; terminal domain (TD), blue; EreA-specific region, purple; A-rich tail, white; PAS, green; and TCTTT terminator, red. (**B**) Diagram of putative amplification dynamics of Ere subfamilies in equid evolution. Diagram shows amplification dynamics of Ere subfamilies in equid evolution, with graph height indicating amplification rate of Ere subfamilies at given time period. Amplification dynamics were inferred based on average sequence similarity in each subfamily (Figure 2) and on number of polymorphic copies of different subfamilies found in horse and zebra genomes. SINE class (T+ or T^−^) of Ere subfamilies is indicated on left of diagram.

This study provides a detailed analysis of the Ere SINE family in the genomes of horses and other odd-toed ungulates. Ere can be divided into two subfamilies, A and B. Subfamily A is characterized by a specific ~60 bp sequence (marked in purple in Figure 8A). Each subfamily can be further divided into two based on the presence and number of a 50 bp sequence (terminal domain, TD) in the 3′-end of these SINEs (highlighted in blue in Figure 8A). This sequence is absent in EreA_0TD; EreA_1TD and EreB_1TD each have a single copy of TD, whereas EreB_2TD has two tandemly located TDs. TDs and the rest of Ere are spaced by short A-rich linkers, indicating that the EreA_1TD, EreB_1TD, and EreB_2TD subfamilies could have arisen from the attachment of TD to the poly(A) tail of the SINE precursor and the successful propagation of the resulting SINEs in the genomes of ancient ungulates. TD sequences in RNA molecules can form long hairpin structures with a double-helical stem up to 13 bp long and a loop consisting of 7–8 nucleotides (Figure 3 and Figure S3). These relatively conserved structures within transcripts could favor the retrotransposition of EreA_1TD, EreB_1TD, and EreB_2TD contributing to the evolutionary success of these SINEs. These three Ere subfamilies are present in the genomes of all families of ungulate species, including equids, rhinos, and tapirs. The number of EreA_1TD copies in tapirs was four times lower than in the genomes of horses and rhinoceroses. Additionally, tapir genomes contain numerous copies of tapir-specific EreB subfamilies, designated as EreB’_1TD and EreB’_2TD (Figure 1 and Figure 2). It appears that subfamilies EreA_1TD, EreB_1TD, and EreB_2TD arose before the separation of ungulates into equids, rhinoceroses, and tapirs, while subfamilies EreB’_1TD and EreB’_2TD emerged in tapirs after their separation. EreB’_1TD might have evolved from EreB_1TD and gave rise to EreB’_2TD. Judging by the average sequence similarity of copies (Figure 2), it can be inferred that EreB_1TD and EreA’_0TD are the oldest subfamilies. It is likely that recombination events between their sequences gave rise to other subfamilies (EreA’_1TD, EreA_1TD, EreA_2TD, and EreA_0TD).

All Ere subfamilies containing TD belong to T^−^ SINEs, whereas the EreA_0TD subfamily should be assigned to the T^+^ class due to the presence of PAS (AATAAA) and the pol III transcription terminator (Figure 1 and Figure 7). EreA_0TD copies from equid genomes contain a single PAS, while copies of EreA_0TD from rhinoceroses have an average of three tandemly located PASs. T^+^ SINEs usually contain multiple PASs, although Ves from bat genomes are an example of SINEs with a single PAS [19,33]. The equid EreA_0TD is characterized by the TCTTT terminator. It can be found in approximately 45 and 70% of copies of the old (EreA_0TD_a) and young (EreA_0TD_b) variants of this SINE, respectively (Figure 4B). The TCTTT terminator is classified as moderately effective, stopping pol III transcription with a 50% probability [33,50]. The prevalence of the rudimentary TCTT terminator among EreA_0TD copies (20–40%) (Figure 4B) confirms that pol III transcription was halted at the TCTTT, resulting in a TCTTT-to-TCTT conversion in the daughter copy. The TCTTT terminator is also prevalent in rhino EreA_0TD copies (40 and 70% in black and white rhinoceroses, respectively). The TTATT and TTAT sequences are often found in place of the terminator, particularly in black rhinoceros. These may be rudiments of the functional TTATTT terminator (Figure 5B). It is worth noting that tapir genomes have 50 times fewer copies of the EreA_0TD subfamily than horses and rhinos. The EreA_0TD copies in tapirs belong to the T^−^ SINE class, which may explain their low retrotranspositional activity.

We searched for and analyzed polymorphic Ere copies present in the genome of one ungulate species that were absent from orthologous loci in another species. Polymorphic copies are relatively young because they arose and integrated into the genome after the divergence of the two species. Different equid species have between 9000 and 14,000 polymorphic copies of Ere in their genomes. The smallest number of copies (1.5–2.5 thousand) is found in the genomes of domestic and Przewalski horses, which is likely due to the recent divergence of these two species 45,000 years ago. Other researchers have also observed high Ere retropositional activity in horses, which peaked about 500,000 years ago [51]. The analysis of polymorphic copies belonging to different subfamilies of Ere shows that the vast majority of them (90–95%) belong to the subfamily EreA_0TD. A much smaller proportion (5–8%) is represented by EreB_2TD copies, and polymorphic copies of the other two subfamilies (EreA_1TD and EreB_1TD) occur in even smaller numbers. Thus, in equid evolution, EreA_0TD (T^+^ SINE) was the most retrotranspositionally active subfamily in the recent millions of years, while EreB_2TD, EreB_1TD, and EreA_1TD (T^−^ SINEs) were the least active subfamilies. The three latter subfamilies were active in the earlier evolution of equids and other ungulates. Figure 8B illustrates the expected amplification dynamics of the four Ere subfamilies in equid genomes. Similar amplification dynamics occurred in the lineage of the white rhinoceros. The EreA_0TD subfamily is the most retrotranspositionally active, whereas EreB_2TD is 8-fold less active (Table 2). Interestingly, the pattern is significantly different in the lineage of the black rhinoceros: the EreB_2TD subfamily is 1.5-fold more active than EreA_0TD (Table 2). Therefore, the behavior of the two SINE subfamilies may differ significantly in different rhino genera (*Ceratotherium* and *Diceros*). Finally, the comparison of the genomes of tapirs *T. indicus* and *T. terrestris* revealed that the tapir-specific EreB’_2TD subfamily remained almost the only active subfamily in these lineages for the last 23 million years.

**Figure 9:**
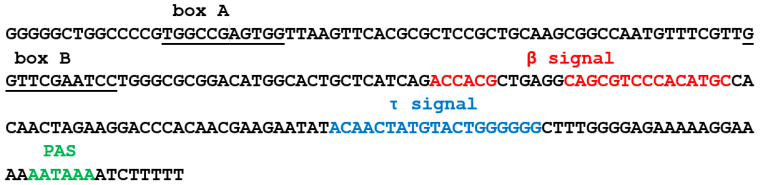
Nucleotide sequence of Ere_0TD copy with identified sites required for efficient polyadenylation of Ere transcripts synthesized by pol III. β, τ, and PAS signals are shown in red, blue, and green, respectively. β signal consists of two parts separated by six nucleotides. Boxes A and B of pol III promoter are underlined.

Experiments were conducted with a single copy of EreA_0TD from the horse genome. Numerous constructs were generated with deletions or substitutions, and they were subsequently transfected into HeLa cells. This allowed us to identify sequences critical for the polyadenylation of pol III transcripts from this SINE. In addition to the PAS, two sites were identified in EreA_0TD (Figure 9) that had both similarities and distinctions from the β and τ signals of T^+^ SINEs studied previously. The τ signal in Ere is similar to that in B2 in terms of localization and length, and it also contains TGTA, a binding site for the auxiliary polyadenylation factor CFIm in B2 RNA [19]. A unique feature of τ signaling in Ere is that the deletion of TGTA alone had almost no effect on polyadenylation, but the deletion of all 19 nucleotides of the τ signal significantly reduced it. Additionally, we did not find any sites consisting of six G residues in the τ signal in other SINEs previously. The β signal in B2, Dip, and Ves SINEs is located five nucleotides away from the B box of the pol III promoter. In contrast, the β signal in Ere is separated from the B box by a much longer (28 nt) sequence. The Ere β signal contains a CCCACATGC site, which is similar to the ACCACATGG sequence previously identified as the β signal of B2 [19]. These sequences are expected to function similarly. However, the Ere β signal was found to be considerably longer, comprising two 6 and 15 nt segments separated by a 6-nucleotide spacer (Figure 9). It is probable that multiple proteins bind the Ere β signal in RNA to promote polyadenylation.

## 5. Conclusions

In conclusion, the SINE Ere family is of ancient origin. It emerged in a common ancestor of odd-toed ungulates estimated to be 60–65 million years old [47,52]. The majority of Ere copies belong to T^−^ SINEs, and they amplified in the genomes of all three families of ungulates (equids, rhinoceroses, and tapirs) throughout the long evolutionary history of these mammals. Later, the retrotransposition of the existing Ere copies was greatly reduced or stopped completely. In tapirs, a distinct subfamily, EreB’_2TD, also a T^−^ SINE, emerged and became the only active Ere subfamily in their genomes. The EreA_0TD subfamily, belonging to T^+^ SINEs, likely arose independently in horses and rhinoceroses. EreA_0TD copies are distinct from other Ere subfamilies as they possess a PAS and a pol III transcription terminator, and their transcripts have been experimentally reported to be polyadenylation competent. EreA_0TD copies, capable of retrotransposition by the T^+^ mechanism, formed the Ere subfamily most actively amplified in equid and rhinoceros genomes during the last few million years of their evolution.

## Figures and Tables

**Table 1:** Number of EreA and EreB copies present in genome of one equid species (+) but absent in orthologous loci of another species (−).

	SINE Family	Domestic horse (−)	Przewalski horse (−)	Domestic donkey (−)	Plains zebra (−)
Domestic horse (+)	EreA		2241	12,592	11,496
	EreB		204	1974	600
Przewalski horse (+)	EreA	1301		nd	8961
	EreB	199		nd	552
Domestic donkey (+)	EreA	8491	nd		nd
	EreB	438	nd		nd
Plains zebra (+)	EreA	10,155	10,164	nd	
	EreB	564	587	nd	

nd, not determined.

**Table 2:** Number of EreA and EreB copies present in genome of one rhinoceros or tapir species (+) but absent in orthologous loci of another species (−) and distribution of polymorphic copies by Ere subfamilies.

	Black rhinoceros (−)	White rhinoceros (−)	Malayan tapir (−)	Lowland tapir (−)
Black rhinoceros (+)		**25,373** EreA_0TD: 39% EreA_1 and 2TD: 4% EreB_2TD: 57%		
White rhinoceros (+)	**30,285** EreA_0TD: 87% EreA_1 and 2TD: 2% EreB_2TD: 11%			
Malayan tapir (+)				**25,754** EreB’_2TD: 100%
Lowland tapir (+)			**10,941** EreB’_2TD: 100%	

## Data Availability

Data are contained within the article and in the Supplementary Materials.

## Supplementary Materials

The following supporting information can be downloaded at https://www.mdpi.com/article/10.3390/ani14131982/s1, Figure S1: The nucleotide sequences of a Ere_0TD copy from the domestic horse genome (Ere-T construct) and a Sor SINE copy from the shrew genome (*Sorex araneus*); Figure S2: The similarity of the Ere 5′-terminal part with tRNA^Glu^_CTC_; Figure S3: Predicted secondary structure of terminal domain (TD) of Ere SINE transcripts from of *Malayan tapir*; Figure S4: Nucleotide sequences of Ere_0TD copies of tribe #1; Figure S5: The distribution of pol III terminators or their rudiments among the 471 tribe #1 copies of EreA_0TD in the domestic horse genome; Figure S6: Effect of deletions in putative τ region of Ere on polyadenylation of hybrid Sor/Ere transcripts; Figure S7. Selected Northern hybridization data obtained in HeLa transfection experiments; Figure S8. Northern hybridization analysis for HeLa cells transfected with Ere constructs carrying deletions or nucleotide substitutions upstream of the β region.

## References

[1] Kramerov D.A., Vassetzky N.S. (2005). Short retroposons in eukaryotic genomes. Int. Rev. Cytol..

[2] Deininger P. (2011). Alu elements: Know the SINEs. Genome Biol..

[3] Kramerov D.A., Vassetzky N.S. (2011). SINEs. Wiley Interdiscip. Rev. RNA.

[4] Chen J.M., Ferec C., Cooper D.N. (2006). LINE-1 endonuclease-dependent retrotranspositional events causing human genetic disease: Mutation detection bias and multiple mechanisms of target gene disruption. J. Biomed. Biotechnol..

[5] Kosushkin S.A., Ustyantsev I.G., Borodulina O.R., Vassetzky N.S., Kramerov D.A. (2022). Tail Wags Dog’s SINE: Retropositional Mechanisms of Can SINE Depend on Its A-Tail Structure. Biology.

[6] Ferrigno O., Virolle T., Djabari Z., Ortonne J.P., White R.J., Aberdam D. (2001). Transposable B2 SINE elements can provide mobile RNA polymerase II promoters. Nat. Genet..

[7] Su M., Han D., Boyd-Kirkup J., Yu X., Han J.J. (2014). Evolution of Alu elements toward enhancers. Cell Rep..

[8] Policarpi C., Crepaldi L., Brookes E., Nitarska J., French S.M., Coatti A., Riccio A. (2017). Enhancer SINEs Link Pol III to Pol II Transcription in Neurons. Cell Rep..

[9] Krull M., Brosius J., Schmitz J. (2005). Alu-SINE exonization: En route to protein-coding function. Mol. Biol. Evol..

[10] Wang W., Kirkness E.F. (2005). Short interspersed elements (SINEs) are a major source of canine genomic diversity. Genome Res..

[11] Krane D.E., Hardison R.C. (1990). Short interspersed repeats in rabbit DNA can provide functional polyadenylation signals. Mol. Biol. Evol..

[12] Chen C., Ara T., Gautheret D. (2009). Using Alu elements as polyadenylation sites: A case of retroposon exaptation. Mol. Biol. Evol..

[13] Carrieri C., Cimatti L., Biagioli M., Beugnet A., Zucchelli S., Fedele S., Pesce E., Ferrer I., Collavin L., Santoro C. (2012). Long non-coding antisense RNA controls Uchl1 translation through an embedded SINEB2 repeat. Nature.

[14] Sharma H., Valentine M.N.Z., Toki N., Sueki H.N., Gustincich S., Takahashi H., Carninci P. (2024). Decryption of sequence, structure, and functional features of SINE repeat elements in SINEUP non-coding RNA-mediated post-transcriptional gene regulation. Nat. Commun..

[15] Ponicsan S.L., Kugel J.F., Goodrich J.A. (2010). Genomic gems: SINE RNAs regulate mRNA production. Curr. Opin. Genet. Dev..

[16] Zovoilis A., Cifuentes-Rojas C., Chu H.P., Hernandez A.J., Lee J.T. (2016). Destabilization of B2 RNA by EZH2 Activates the Stress Response. Cell.

[17] Borodulina O.R., Kramerov D.A. (2001). Short interspersed elements (SINEs) from insectivores. Two classes of mammalian SINEs distinguished by A-rich tail structure. Mamm. Genome.

[18] Vassetzky N.S., Kramerov D.A. (2013). SINEBase: A database and tool for SINE analysis. Nucleic Acids Res..

[19] Ustyantsev I.G., Borodulina O.R., Kramerov D.A. (2021). Identification of nucleotide sequences and some proteins involved in polyadenylation of RNA transcribed by Pol III from SINEs. RNA Biol..

[20] Borodulina O.R., Kramerov D.A. (2008). Transcripts synthesized by RNA polymerase III can be polyadenylated in an AAUAAA-dependent manner. RNA.

[21] Borodulina O.R., Golubchikova J.S., Ustyantsev I.G., Kramerov D.A. (2016). Polyadenylation of RNA transcribed from mammalian SINEs by RNA polymerase III: Complex requirements for nucleotide sequences. Biochim. Biophys. Acta.

[22] Proudfoot N.J. (2011). Ending the message: Poly(A) signals then and now. Genes Dev..

[23] Sun Y., Hamilton K., Tong L. (2020). Recent molecular insights into canonical pre-mRNA 3′-end processing. Transcription.

[24] Liu J., Lu X., Zhang S., Yuan L., Sun Y. (2022). Molecular Insights into mRNA Polyadenylation and Deadenylation. Int. J. Mol. Sci..

[25] Boreikaite V., Passmore L.A. (2023). 3′-End Processing of Eukaryotic mRNA: Machinery, Regulation, and Impact on Gene Expression. Annu. Rev. Biochem..

[26] Borodulina O.R., Ustyantsev I.G., Kramerov D.A. (2023). SINEs as Potential Expression Cassettes: Impact of Deletions and Insertions on Polyadenylation and Lifetime of B2 and Ves SINE Transcripts Generated by RNA Polymerase III. Int. J. Mol. Sci..

[27] Roy-Engel A.M., Salem A.H., Oyeniran O.O., Deininger L., Hedges D.J., Kilroy G.E., Batzer M.A., Deininger P.L. (2002). Active Alu element “A-tails”: Size does matter. Genome Res..

[28] Odom G.L., Robichaux J.L., Deininger P.L. (2004). Predicting mammalian SINE subfamily activity from A-tail length. Mol. Biol. Evol..

[29] Dewannieux M., Heidmann T. (2005). Role of poly(A) tail length in Alu retrotransposition. Genomics.

[30] Roy-Engel A.M. (2012). A tale of an A-tail: The lifeline of a SINE. Mob. Genet. Elem..

[31] Batzer M.A., Deininger P.L. (2002). Alu repeats and human genomic diversity. Nat. Rev. Genet..

[32] Wagstaff B.J., Hedges D.J., Derbes R.S., Campos Sanchez R., Chiaromonte F., Makova K.D., Roy-Engel A.M. (2012). Rescuing Alu: Recovery of new inserts shows LINE-1 preserves Alu activity through A-tail expansion. PLoS Genet..

[33] Vassetzky N.S., Borodulina O.R., Ustyantsev I.G., Kosushkin S.A., Kramerov D.A. (2021). Analysis of SINE Families B2, Dip, and Ves with Special Reference to Polyadenylation Signals and Transcription Terminators. Int. J. Mol. Sci..

[34] Sakagami M., Ohshima K., Mukoyama H., Yasue H., Okada N. (1994). A novel tRNA species as an origin of short interspersed repetitive elements (SINEs): Equine SINEs may have originated from tRNA(Ser). J. Mol. Biol..

[35] Gallagher P.C., Lear T.L., Coogle L.D., Bailey E. (1999). Two SINE families associated with equine microsatellite loci. Mamm. Genome.

[36] Santagostino M., Khoriauli L., Gamba R., Bonuglia M., Klipstein O., Piras F.M., Vella F., Russo A., Badiale C., Mazzagatti A. (2015). Genome-wide evolutionary and functional analysis of the Equine Repetitive Element 1: An insertion in the myostatin promoter affects gene expression. BMC Genet..

[37] Yamada K.D., Tomii K., Katoh K. (2016). Application of the MAFFT sequence alignment program to large data-reexamination of the usefulness of chained guide trees. Bioinformatics.

[38] Pearson W.R., Lipman D.J. (1988). Improved tools for biological sequence comparison. Proc. Natl. Acad. Sci. USA.

[39] Li H. (2013). Aligning sequence reads, clone sequences and assembly contigs with BWA-MEM. arXiv.

[40] Shen W., Le S., Li Y., Hu F. (2016). SeqKit: A Cross-Platform and Ultrafast Toolkit for FASTA/Q File Manipulation. PLoS ONE.

[41] Quinlan A.R., Hall I.M. (2010). BEDTools: A flexible suite of utilities for comparing genomic features. Bioinformatics.

[42] Chomczynski P., Sacchi N. (2006). The single-step method of RNA isolation by acid guanidinium thiocyanate-phenol-chloroform extraction: Twenty-something years on. Nat. Protoc..

[43] MacHugh D.E., Larson G., Orlando L. (2017). Taming the Past: Ancient DNA and the Study of Animal Domestication. Annu. Rev. Anim. Biosci..

[44] Der Sarkissian C., Ermini L., Schubert M., Yang M.A., Librado P., Fumagalli M., Jonsson H., Bar-Gal G.K., Albrechtsen A., Vieira F.G. (2015). Evolutionary Genomics and Conservation of the Endangered Przewalski’s Horse. Curr. Biol..

[45] Orlando L., Ginolhac A., Zhang G., Froese D., Albrechtsen A., Stiller M., Schubert M., Cappellini E., Petersen B., Moltke I. (2013). Recalibrating Equus evolution using the genome sequence of an early Middle Pleistocene horse. Nature.

[46] Moodley Y., Westbury M.V., Russo I.M., Gopalakrishnan S., Rakotoarivelo A., Olsen R.A., Prost S., Tunstall T., Ryder O.A., Dalen L. (2020). Interspecific Gene Flow and the Evolution of Specialization in Black and White Rhinoceros. Mol. Biol. Evol..

[47] Liu S., Westbury M.V., Dussex N., Mitchell K.J., Sinding M.S., Heintzman P.D., Duchene D.A., Kapp J.D., von Seth J., Heiniger H. (2021). Ancient and modern genomes unravel the evolutionary history of the rhinoceros family. Cell.

[48] Humphreys A.M., Barraclough T.G. (2014). The evolutionary reality of higher taxa in mammals. Proc. Biol. Sci..

[49] Lim Q.L., Yong C.S.Y., Ng W.L., Ismail A., Rovie-Ryan J.J., Rosli N., Annavi G. (2021). Genetic diversity and phylogenetic relationships of Malayan tapir (*Tapirus indicus*) populations in the Malay Peninsula based on mitochondrial DNA control region. Biodivers. Conserv..

[50] Orioli A., Pascali C., Quartararo J., Diebel K.W., Praz V., Romascano D., Percudani R., van Dyk L.F., Hernandez N., Teichmann M. (2011). Widespread occurrence of non-canonical transcription termination by human RNA polymerase III. Nucleic Acids Res..

[51] Liu X., Zhang Y., Pu Y., Ma Y., Jiang L. (2023). Whole-genome identification of transposable elements reveals the equine repetitive element insertion polymorphism in Chinese horses. Anim. Genet..

[52] Secord R., Bloch J.I., Chester S.G., Boyer D.M., Wood A.R., Wing S.L., Kraus M.J., McInerney F.A., Krigbaum J. (2012). Evolution of the earliest horses driven by climate change in the Paleocene-Eocene Thermal Maximum. Science.

