# Supplementary material for: Ere, a Family of Short Interspersed Elements in the Genomes of Odd-Toed Ungulates (Perissodactyla)
Source: Animals (Basel). 2024 Jul 5;14(13):1982. doi: 10.3390/ani14131982 (PMC11240701; doi:10.3390/ani14131982)
Supplement: Supplementary file 1 [file animals-14-01982-s001.zip › Figures S1-S8 pdf/Figure S1.pdf]

Ere_T construct. GenBank: AC124156.3; position 85175-85438 (equCab3/Horse, chr4:74155812-74156088)

catagaatggttaaaggatgtagaaagctatggaataaaagagacaaactGGGGGCTGGCCCCGTGGCCGAGTGGTTAAGTTCACGCGCTCCGCTGCAAGCGGCCAATGT
TTCGTTGGTTCGAATCCTGGGCGCGGACATGGCACTGCTCATCAGACCACGCTGAGGCAGCGTCCCACATGCCACAACTAGAAGGACCCACAACGAAGAATATACAACTA
TGTACTGGGGGGCTTTGGGGAGAAAAAGGAAAA**AATAAA**ATCTTTTT

*

AACAAA (Ere_C construct)

SOR_T, clone Sar 4SA, GenBank: AF195925

tatatggcaacccaacctctttgttagtaaacattcagtgtaatcttggtgcagtctttacaaaatcttttcatcccttttttcttcataaaaatagacatcacgggGGGC
TGGAGTGATAGCACAGTGGGTAGGGCGTTTGCCTTGCATGCGACCGACCCGGGTTCGAATCCCAGCATCCCATATGGTCCTCTGAGCACCGCCAGGAGTGATTCCTAAGT
GCAGAGCCAGGAGTAACCCCTGTGCATTGCCAGGTGTGACCCAAAAAGCAAAAAAAAAAAAA

SOR/Ere_T

tatatggcaacccaacctctttgttagtaaacattcagtgtaatcttggtgcagtctttacaaaatcttttcatcccttttttcttcataaaaatagacatcacgggGGGC
TGGAGTGATAGCACAGTGGGTAGGGCGTTTGCCTTGCATGCGACCGACCCGGGTTCGAATCCCAGCATCCCATATGGTCCTCTGAGCACCGCCAGGAGTGATTCCTAAGT
GCAGAGCCAGGAGTAACCCCTGTGCATTGCCAGGTGTGACCC*AGATCT*TAGAAGGACCCACAACGAAGAATATACAACTATGTACTGGGGGGCTTTGGGGAGAAAAAGGA
AAA**AATAAA**ATCTTTTT

Bgl II

Figure S1. Nucleotide sequences of a Ere_0TD copy from the domestic horse genome (Ere_T construct) and a Sor SINE copy from the shrew genome (*Sorex araneus*), which were used to identify β and τ signals in HeLa cell transfection experiments. The 5'-flanking sequences are given in lowercase letters, and the SINE sequences are represented in capital letters (Ere and Sor sequences are highlighted in yellow and blue, respectively). Boxes A and B of the pol III promoters are underlined. PAS is shown in bold. The Ere_C construct differs from Ere_T only by a T to C substitution in PAS. Two T residues were added to the transcription terminator of the Ere-T construct (TCTTTTT) to increase its efficiency. The *Bgl* II site, which was introduced into the Ere and Sor sequences for cloning into the Sor/Ere_T construct, is italicized. The first nucleotide of the Ere_T fragment transferred into the Sor/Ere_T construct is marked with an asterisk.
