# Supplementary material for: Ere, a Family of Short Interspersed Elements in the Genomes of Odd-Toed Ungulates (Perissodactyla)
Source: Animals (Basel). 2024 Jul 5;14(13):1982. doi: 10.3390/ani14131982 (PMC11240701; doi:10.3390/ani14131982)
Supplement: Supplementary file 1 [file animals-14-01982-s001.zip › Figures S1-S8 pdf/Figure S2.pdf]

```
>>tdbD00004694|Homo_sapiens|9606|Glu|CTC
(72 nt)
 s-w opt: 179  Z-score: 197.5  bits: 41.1 E(361): 1.3e-006
Smith-Waterman score: 179; 77.1% identity (78.6% similar) in 70 nt overlap (9-76:3-71)

               10        20          30        40          50        60        70        80
90
hoEre  GGGGCCGGCCCSGTGGCCTAGTGGTTAAG-TTCGCGCGCTC-CGCTTCGGCGGCCCGGGTTCGGATCCCGGGCGCGGACMTAGCA
               :: .:::: :::::::::: : :::: :::::: : :  : ::::::::::::::  :::::: :  :::
tRNA_Glu     TCCCTGGTGGTCTAGTGGTTAGGATTCG-GCGCTCTCACCGCCGCGGCCCGGGTTCGATTCCCGGTCAGGGAA
                     10        20          30        40        50        60        70


>>tdbD00004694|Homo_sapiens|9606|Glu|CTC
(72 nt)
 s-w opt: 146  Z-score: 155.2  bits: 33.3 E(361): 0.00029
Smith-Waterman score: 146; 73.4% identity (73.4% similar) in 64 nt overlap (9-69:3-66)

               10         20          30        40        50        60        70        80
rhEre  GGGGCCGGCCCCGTGG-CTAGCGGTTAAG--TGCGCGCGCTCCGCTCGGCGGCCCGGGTTCGGATCCCGGGCGCGCACCGACGC
               ::  :::: :::: ::::: :  :  :::: :::  : : ::::::::::::::  :::::: :
tRNA_Glu     TCCCTGGTGGTCTAGTGGTTAGGATTCGGCGCTCTCACCGCCGCGGCCCGGGTTCGATTCCCGGTCAGGGAA
                     10        20        30        40        50        60        70


>>tdbD00004694|Homo_sapiens|9606|Glu|CTC
 s-w opt: 153  Z-score: 176.8  bits: 37.2 E(361): 1.8e-005
Smith-Waterman score: 153; 75.7% identity (75.7% similar) in 70 nt overlap (9-75:3-71)

               10        20          30         40        50        60        70        80
taEre  GGGGCCGGCCCGGTGGCGTAG-GGTTAAG-TTCGCGCGCTC-CGCTTCGGCGGCCCGGGTTCGGATCCCGGGCGCGGACCTACC
               :: :::::  ::: ::::: : :::: :::::: : :  : ::::::::::::::  :::::: :  :::
tRNA_Glu     TCCCTGGTGGTCTAGTGGTTAGGATTCG-GCGCTCTCACCGCCGCGGCCCGGGTTCGATTCCCGGTCAGGGAA
                     10        20          30        40        50        60        70
```

Figure S2. Similarity of the Ere 5'-terminal part with $tRNA^{Glu}_{CTC}$. Consensus sequences of domestic horse (hoEre), black rhinoceros (rhEre) and Malayan tapir (taEre) Ere were compared with all human tRNAs. Consensus sequences were generated from all EreA and EreB subfamilies (see Figure 1). The 9-bp insertion (duplication) was removed from the final Ere consensus sequences. Boxes A and B are underlined. $tRNA^{Glu}_{CTC}$ showed the highest similarity to Ere (sequence similarity levels are highlighted in yellow).
