# Supplementary material for: Ere, a Family of Short Interspersed Elements in the Genomes of Odd-Toed Ungulates (Perissodactyla)
Source: Animals (Basel). 2024 Jul 5;14(13):1982. doi: 10.3390/ani14131982 (PMC11240701; doi:10.3390/ani14131982)
Supplement: Supplementary file 1 [file animals-14-01982-s001.zip › Figures S1-S8 pdf/Figure S3.pdf]

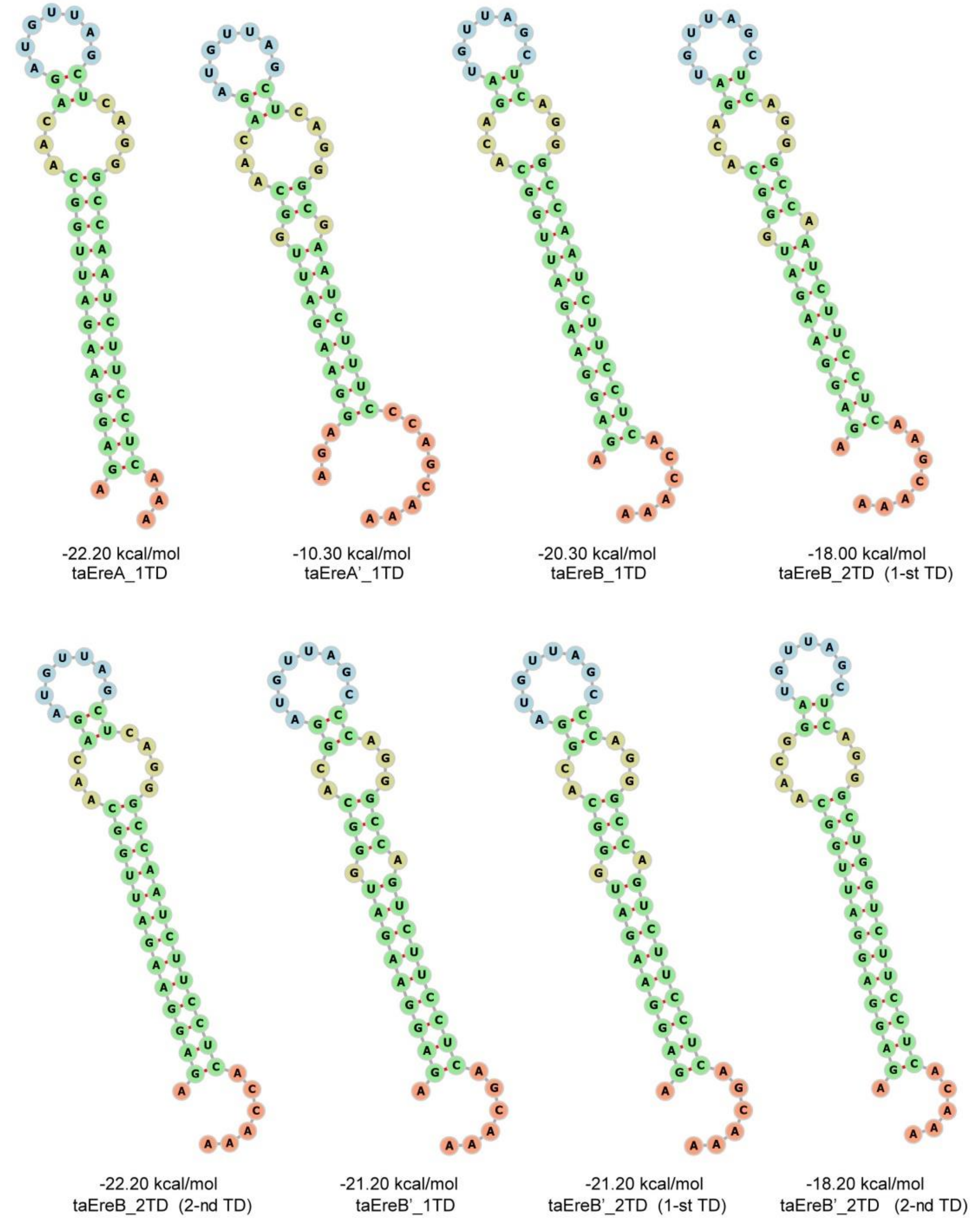


**Figure S3.** Predicted secondary structure of terminal domain (TD) of Ere SINE transcripts from of Malayan tapir. The consensus sequences depicted in Figure 1C were folded. Their free energy values and the corresponding Ere subfamilies are indicated below the structures. Blue indicates loop nucleotides, while green and yellow highlight paired and unpaired stem nucleotides, respectively.
