# Supplementary material for: Ere, a Family of Short Interspersed Elements in the Genomes of Odd-Toed Ungulates (Perissodactyla)
Source: Animals (Basel). 2024 Jul 5;14(13):1982. doi: 10.3390/ani14131982 (PMC11240701; doi:10.3390/ani14131982)
Supplement: Supplementary file 1 [file animals-14-01982-s001.zip › Figures S1-S8 pdf/Figure S4.pdf]

```
tribe#1                          -----------------------GGGGCTGGCCCCGTGGCCGAGTGGTTAAGTTCGCGCGCTCCGCTGCAGGCGGCCCAGTGTTTCGTCGGTTCGAATCCTGGGCGCGGACATGGCACTGCTCGTCAGACCACGCTGAGGCAGCGTCCCACATGCCACAACT--AGAGGA
CM009148.1:138772419-138772829   ATGAGCAAGAAAGAACAAATCCTGGGGCTGGCCCCGTGGCCGAGTGGTTAAGTTCGCGCGCTCCGCTGCAGGCGGCCCAGTGTTTCGTCGGTTCGAATCCTGGGCGCGGACATGGCACTGCTCGTCAGACCACGCTGAGGCAGCGTCCCACATGCCACAACT--AGAGGA
CM009148.1:156178634-156179057   TAATCTTAAAAATGGTTGTGTGGGGGGCTGGCCCCGTGGCCGAGTGGTTAAGTTCGCGCGCTCCGCTGCAGGCGGCCCAGTGTTTCGTCGGTTCGAATCCTGGGCGCGGACATGGCACTGCTCGTCAGACCACGCTGGGGCAGCGTCCCACATGCCACAACT--AGAGGA
CM009148.1:177304996-177305407   TTCTCAAAAAGTTACACATAAATGGGGCTGGCCCCGTGGCCGAGTGGTTAAGTTCGCGCGCTCCGCTGCAGGCGGCCCAGTGTTTCGTCGGTTCGAATCCTGGGCGCGGACATGGCACTGCTCGTCAGACCACGCTGAGGCAGCGTCCCACATGCCACAACT--AGAGGA
CM009148.1:42502200-42502617     CTGGATTAAAAACTGGTTTTCTTGGGGCTGGCCCCGTGGCCGAGTGGTTAAGTTCGCGCGCTCTGCTGCAGGCGGCCCAGTGTTTCGTCGGTTCGAATCCTGGGCGCGGACATGGCACTGCTCGTCAGACCACGCTGAGGCAGCGTCCCACATGCCACAACT--AGAGGA
CM009148.1:50345615-50346026     TTAGGCAAAGAGAGTAAGATCAAGGGGCTGGCCCCGTGGCCGAGTGGTTAAGTTCGCGCGCTCCGCTGCAGGCGGCCCAGTGTTTCGTCGGTTCGAATCCTGGGCGCGGACATGGCACTGCTCGTCAGACCACGCTGAGGCAGCGTCCCACATGCCACAACT--AGAGGA
CM009148.1:83211467-83211877     TGAAATAAAAAAACACTTATCTAGGGGCTGGCCCCGTGGCCGAGTGGTTAAGTTCGCGCGCTCCGCTGCAGGCGGCCCAGTGTTTCGTCGGTTCGAATCCTGGGCGCGGACATGGCACTGCTCGTCAGACCACGCTGAGGCAGCGTCCCACATGCCACAACT--AGAGGA
CM009148.1:97375328-97375750     AACTGCTTAAAAGGTAGATACGGGGGGCTGGCCCTGTGGCCGAGTGGTTAAGTTCGCGCGCTCCGCTGCAGGCGGCCCAGTGTTTCGTCGGTTCGAATCCTGGGCGCGGACATGGCACTGCTCGTCAGACCACGCTGAGGCAGCGTCCCACATGCCACAACT--AGAGGA
CM009149.1:55409213-55409682     TTTTTAAATTAAGAAATAATTGAGGGGCTGGCCCCGTGGCCGAGTGGTTAAGTTCGCGCGCTCCGCTGCAGGCAGCCCAGTGTTTCGTCGGTTCGAATCCTGGGCGCGGACATGGCACTGCTCGTCAGACCACGCTGAGGCAGCGTCCCACATGCCACAACT--AGAGGA
CM009149.1:65055069-65055492     TTTATTCTTAGAAAGTGGGTGTTGGGGCTGGCCCCGTGGCCGAGTGGTTAAGTTCGCGCGCTCCGCTGCAGGCGGCCCAGTGTTTCGTCGGTTCGAATCCTGGGCGCGGACATGGCACTGCTCGTCAGACCACGCTGAGGCAGCGTCCCACATGCCACAACT--AGAGGA
CM009150.1:21874158-21874568     GACACTTATAAAAACAGATTTCAGGGGCTGGCCCCGTGGCCGAGTGGTTAAGTTCGCGCGCTCCGCTGCAGGCGGCCCAGTGTTTCGTCGGTTCGAATCCTGGGCGCGGACATGGCACTGCTCGTCAGACCACGCTGAGGCAGCGTCCCACATGCCACAACT--AGAGGA
CM009150.1:56707839-56708282     TCGCAAAATTGCATCAGTATATTGGGGCTGGCCCCGTGGCCGAGTGGTTAAGTTCGCGCGCTCCGCTGCAGGCGGCCCAGTGTTTCGTCGGTTCGAATCCTGGGCGCGGACATGGCACTGCTCGTCAGACCACGCTGAGGCAGCGTCCCACATGCCACAACT--AGAGGA
CM009150.1:89781678-89782145     TGTTCTTTAAAAGACCCAAATCTGGGGCTGGCCCCGTGGCCGAGTGGTTAAGTTCGCGCGCTCCGCTGCAGGCGGCCCAGTGTTTCGTCGGTTCGAATCCTGGGCGCGGACATGGCACTGCTCGTCAGACCACGCTGAGGCAGCGTCCCACATGCCACAACT--AGAGGA
CM009151.1:10189497-10189907     TAATTAAAAAATAAAAAAAGGATGGGGCTGGCCCCGTGGCCGAGTGGTTAAGTTCGCGCGCTCCGCTGCAGGCGGCCCAGTGTTTCGTCGGTTCGAATCCTGGGCGCGGACATGGCACTGCTCGTCGGACCACGCTGAGGCAGCGTCCCACATGCCACAACT--AGAGGA
CM009151.1:33498246-33498720     TAACATTTAAAAATAAGTTGCATGGGGCTGGCCCCGTGGCCGAGTGGTTAAGTTCGCGCGCTCCGCTGCAGGCGGCCCAGTGTTTCGTCGGTTCGAATCCTGGGCGCGGACATGGCACTGCTCGTCAGACCACGCTGAGGCAGCGTCCCACATGCCACAACT--AGAGGA
CM009151.1:34742422-34742832     AAGCCTTTTAAGAATCCAAATCTGGGGCTGGCCCCGTGGCCGAGTGGTTAAGTTCGCGCGCTCCGCTGCAGGCGGCCCAGTGTTTCGTCGGTTCGAATCCTGGGCGCGGACATGGCACTGCTCGTCAGACCACGCTGAGGCAGCGTCCCACATGCCACAACT--AGAGGA
CM009151.1:62375983-62376393     GTGCTAATAAGAAGTGTTTTATGGGGGCTGGCCCCGTGGCCGAGTGGTTAAGTTCGCGCGCTCCGCTGCAGGCGGCCCAGTGTTTCGTCGGTTCGAATCCTGGGCGCGGACATGGCACTGCTCGTCAGACCACGCTGAGGCAGCCTCCCACATGCCACAACT--AGAGGA
CM009151.1:63408934-63409348     ACTTCTTTTAAATATATATTGGAGGGGCTGGCCCCGTGGCCGAGTGGTTAAGTTCGCGCGCTCCGCTGCAGGCGGCCCAGTGTTTCGTCGGTTCGAATCCTGGGCGCGGACATGGCACTGCTCGTCAGACCACGCTGAGGCAGCGTCCCACATGCCACAACT--AGAGGA
CM009151.1:77547749-77548159     CTTTAAAGAAGAAGCTAATTTAGGGGGCTGGCCCCGTGGCCGAGTGGTTAAGTTCGCGCGCTCCGCTGCAGGCGGCCCAGTGTTTCGTCGGTTCGAATCCTGGGCGCGGACATGGCACTGCTCGTCAGACCATGCTGAGGCAGCGTCCCACATGCCACAACT--AGAGGA
CM009151.1:92510263-92510678     AATTTTTAAAAAATCCACTGGAAGGGGCTGGCCCCGTGGCCGAGTGGTTAAGTTCGCGCGCTCCGCTGCAGGCGGCCCAGTGTTTCATCGGTTCGAATCCTGGGCGCGGACATGGCACTGCTCGTCAGACCACGCTGAGGCAGCGTCCCACATGCCACAACT--AGAGGA
CM009152.1:75359342-75359817     TGGATTAAAAGTTAACTAAGGCAGGGGCTGGCCCCGTGGCCGAGTGGTTAAGTTCGCGCGCTCCGCTGCAGGCGGCCCAGTGTTTCGTCGGTTCGAATCCTGGGCGCGGACATGGCACTGCTCGTCAGACCACGCTGAGGCAGCGTCCCACATGCCACAACT--AGAGGA
CM009152.1:86760014-86760429     CTTTCTCAAGAAAATATACAAGAGGGGCTGGCCCCGTGGCCGAGTGGTTAAGTTCGCGCGCTCCGCTGCAGGCGGCCCAGTGTTTCATCGGTTCGAATCCTGGGCGCGGACATGGCACTGCTCGTCAGACCACGCTGAGGCAGCGTCCCACATGCCACAACT--AGAGGA
CM009152.1:90931209-90931632     GCCTTCAAGAAACACACAGTCTAGGGGCTGGCCCCGTGGCCGAGTGGTTAAGTTCGCGCGCTCCGCTGCAGGCGGCCCAGTGTTTCGTCGGTTCGAATCCTGGGCGCGGACATGGCACTGCTCGTCAGACCGCGCTGAGGCAGCGTCCCACATGCCACAACT--AGAGGA
CM009153.1:14456134-14456548     GCTGACTCAAAAAGAGATTGGTTGGGGCTGGCCCCGTGGCCGAGTGGTTAAGTTCGCGCGCTCTGCTGCAGGCGGCCCAGTGTTTCGTCGGTTCGAATCCTGGGCGCGGACATGGCACTGCTCGTCAGACCACGCTGAGGCAGCGTCCCACATGCCACAACT--AGAGGA
CM009153.1:63144455-63144865     AAGATTTAAAGCGGTGTCTCAGAGGGGCTGGCCCCGTGGCCGAGTGGTTAAGTTCGCGCGCTCCGCTGCAGGCGGCCCAGTGTTTCGTCGGTTCGAATCCTGGGCGCGGACATGGCACTGCTCGTCAGACCACGCTGAGGCAGCGTCCCACATGCCACAACT--AGAGGA
CM009154.1:45113569-45114033     GGGCTCCTTAAGAGTGCTCATGAGGGGCTGGCCCCGTGGCCGAGTGGTTAAGTTCGCGCGCTCCGCTGCAGGCGGCCCAGTGTTTCGTCGGTTCGAATCCTGGGCGCGGACATGGCACTGCTCGTCAGACCACGCTGAGGCAGCGTCCCACATGCCACAACT--AGAGGA
CM009154.1:57327969-57328425     TATTTTAAAATTGATAATTTATGGGGGCTGGCCCCGTGGCCGAGTGGTTAAGTTCGCGCGCTCTGCTGCAGGCGGCCCAGTGTTTCGTCGGTTCGAATCCTGGGCGCGGACATGGCACTGCTCGTCAGACCACGCTGAGGCAGCGTCCCACATGCCACAACT--AGAGGA
CM009154.1:6935451-6935861       GAAGATCAAGAAATGTCTTCTTCGGGGCTGGCCCCGTGGCCGAGTGGTTAAGTTCGCGCGCTCCGCTGCAGGCGGCCCAGTGTTTCGTCGGTTCGAATCCTGGGCGCGGACATGGCACTGCTCGTCAGACCACGCTGAGGCAGCGTCCCACATGCCACAACT--AGAGGA
CM009155.1:18940473-18940910     GATACTAAGATAACAAAGACCTCGGGGCTGGCCCCGTGGCCGAGTGGTTAAGTTCGCGCGCTCTGCTGCAGGCGGCCCAGTGTTTCGTCGGTTCGAATCCTGGGCGCGGACATGGCACTGCTCGTCAGACCACGCTGAGGCAGCGTCCCACATGCCACAACT--AGAGGA
CM009155.1:30484771-30485181     ATATTTAAAATAGTGACCCACCAGGGGCTGGCCCCGTGGCTGAGTGGTTAAGTTCGCGCGCTCCGCTGCAGGCGGCCCAGTGTTTCGTCGGTTCGAATCCTGGGCGCGGACATGGCACTGCTCGTCAGACCACGCTGAGGCAGCGTCCCACATGCCACAACT--AGAGGA
CM009155.1:39197953-39198376     TAAAGCTATTAAGAATATTTGTAGGGGCTGGCCCCGTGGCCGAGTGGTTAAGTTCGCGCGCTCCGCTGCAGGCGGCCCAGTGTTTCGTCGGTTCGAATCCTGGGCGCGGACATAGCACTGCTCGTCAGACCACGCTGAGGCAGCGTCCCACATGCCACAACT--AGAGGA
CM009155.1:39217216-39217653     CCTTTCCCAAGATGTGGGGAGTAGGGGCTGGCCCCGTGGCCGAGTGGTTAAGTTCGCGCGCTCCGCTGCAGGCGGCCCAGTGTTTCGTCGGTTCGAATCCTGGGCGCGGACATGGCACTGCTCGTCAGACCACGCTGAGGCAGCGTCCCACATGCCACAACT--AGAGGA
CM009155.1:68099595-68100009     CAGAAGTAAAAGCAAGTGATGTTGGGGCTGGCCCCGTGGCCGAGTGGTTAAGTTCGCGCGCTCCGCTGCAGGCGGCCCAGTGTTTCGTCGGTTCGAATCCTGGGCGCGGACATGGCACTGCTCGTCAGACCACGCTGAGGCAGCGTCCCACATGCCACAACT--AGAGGA
CM009155.1:75859036-75859446     TGAGCAAAAGAATACCCGTAATTGGGGCTGGCCCCGTGGCCGAGTGGTTAAGTTCGCGCGCTCCGCTGCAGGCGGCCCAGTGTTTCGTCGGTTCGAATCCTGGGCGCGGACATGGCACTGCTCGTCAGACCACGCTGAGGCAGCGTCCCACATGCCACAACT--AGAGGA
CM009155.1:78559054-78559464     TTTTATCAAGAATTTTTATCTTGGGGGCTGGCCCCGTGGCCGAGTGGTTAAGTTCGCGCGCTCCGCTGCAGGCGGCCCAGTGTTTCGTCGGTTCGAATCCTGGGCGCGGACATGGCACTGCTCGTCAGACCACGCTGAGGCAGCGTCCCACATGCCACAACT--AGAGGA
CM009155.1:86410379-86410789     GTTCTAAATGAGTTACAGTGGATGGGGCTGGCCCCGTGGCCGAGTGGTTAAGTTCGCGCGCTCCGCTGCAGGCGGCCCAGTGTTTCGTCGGTTCGAATCCTGGGCGCGGACATGGCACTGCTCGTCAGACCACGCTGAGGCAGCGTCCCACATGCCACAACT--AGAGGA
CM009155.1:88917393-88917803     AAATTTTTAATAAAGTACATATAGGGGCTGGCCCCGTGGCCGAGTGGTTAAGTTCGCGCACTCCGCTGCAGGCGGCCCAGTGTTTCGTCGGTTCGAATCCTGGGCGCGGACATGGCACTGCTCGTCAGACCACGCTGAGGCAGCGTCCCACATGCCACAACT--AGAGGA
CM009157.1:20514577-20515000     TCTCTTAAGAATGTTGGAGACTTGGGGCTGGCCCCGTGGCCGAGTGGTTAAGTTCGCGCGCTCCGCTGCAGGCGGCCCAGTGTTTCGTCGGTTCGAATCCTGGGCGCGGACATGGCACTGCTCATCAGACCACGCTGAGGCAGCGTCCCACATGCCACAACT--AGAGGA
CM009157.1:2560147-2560557       TACTATAAGAATTAAATGAATGGGGGGCTGGCCCCGTGGCCGAGTGCTTAAGTTCGCGCGCTCCGCTGCAGGCGGCCCAGTGTTTCGTCGGTTCGAATCCTGGGCGCGGACATGGCACTGCTCGTCAGACCACGCTGAGGCAGCGTCCCACATGCCACAACT--AGAGGA
CM009157.1:57774641-57775095     CCACTTTATAAATGTTGTATGTAGGGGCTGGCCCCGTGGCCGAGTGGTTAAGTTCGCGCGCTCCGCTGCAGGTGGCCCAGTGTTTCGTCGGTTCGAATCCTGGGCGCGGACATGGCACTGCTCGTCAGACCACGCTGAGGCAGCGTCCCACATGCCACAACT--AGAGGA
CM009157.1:71872104-71872565     AGTAAAGTTAATAATCCAGTCTTGGGGCTGGCCCCGTGGCCGAGTGGTTAAGTTCGCGCGCTCCGCTGCAGGCGGCCCAGTGTTTCGTCGGTTCGAATCCTGGGCGCGGACATGGCACTGCTCGTCAGACCACGCTGAGGCAGCGTCCCACATGCCACAACT--AGAGGA
CM009157.1:74644096-74644527     TGTTAGGTAAAATAAGCTAAACAGGGGCTGGCCCCGTGGCCGAGTGGTTAAGTTCGCGCGCTCCGCTGCAGGCGGCCCAGTGTTTCGTCGGTTCGAATCCTGGGCGCGGACATGGCGCTGCTCGTCAGACCACGCTGAGGCAGCGTCCCACATGCCACAACT--AGAGGA
CM009158.1:28334415-28334825     GGTATAGAAATATAGCTACTCCCGGGGCTGGCCCCGTGGCCGAGTGGTTAAGTTCGCGCGCTCCGCTGCAGGTGGCCCAGTGTTTCGTCGGTTCGAATCCTGGGCGCGGACATGGCACTGCTCGTCAGACCACGCTGAGGCAGCGTCCCACATGCCACAACT--AGAGGA
CM009159.1:17067376-17067803     ACTTTTTAAAAATAGGGTTTTAAGGGGCTGGCCCCGTGGCCGAGTGGTTAAGTTCGCGCGCTCCGCTGCAGGCGGCCCAGTGTTTCGACGGTTCGAATCCTGGGCGCGGACATGGCACTGCTCGTCAGACCACGCTGAGGCAGCGTCCCACATGCCACAACT--AGAGGA
CM009159.1:19835648-19836088     TTTGCTCAAGAAGATGGGTGCCTGGGGCTGGCCCCGTGGCCGAGTGGTTAAGTTCGCGCGCTCCGCTGCAGGCGGCTCAGTGTTTCGTCGGTTCGAATCCTGGGCGCGGACATGGCACTGCTCGTCAGACCACGCTGAGGCAGCGTCCCACATGCCACAACT--AGAGGA
CM009160.1:15862829-15863239     TGTGCTTTATTTAAAGCATCTTAGGGGCTGGCCCCGTGGCCGAGTGGTTAAGTTCGCGCGCTCCGCTGCAGGCGGCCCAGTGTTTCGTCGGTTCGAATCCTGGGCGCGGACATGGCACTGCTCGTCAGACCACGCTGAGGCAGCGTCCCACATGCCACAACT--AGAGGA
CM009160.1:22015007-22015417     CTGTTTAAAGGCAGAAGAAGCGGGGGGCTGGCCCCGTGGCCGAGTGGTTAAGTTCGCGCGCTCCGCTGCAGGTGGCCCAGTGTTTCGTCGGTTCGAATCCTGGGCGCGGACATGGCACTGCTCGTCAGACCACGCTGAGGCAGCGTCCCACATGCCACAACT--AGAGGA
CM009160.1:38725428-38725838     TTGTTTAGAAATATAGAGCTCCTGGGGCTGGCCCCGTGGCCGAGTGGTTAAGTTCGCGCGCTCCGCTGCAGGCGGCCCAGTGTTTCGTCGGTTCGAATCCTGGGCGCGGACATGGCACTGCTCGTCAGACCACGCTGAGGCAGCGTCCCACATGCCACAACT--AGAGGA
CM009161.1:38386804-38387214     ATCATCAGAAATGTAAATATCTGGGGGCTGGCCCCGTGGCCGAGTGGTTAAGTTCGCGCGCTCCGCTGCAGGTGGCCCAGTGTTTCGTCGGTTCGAATCCTGGGCGCGGACATGGCACTGCTCGTCAGACCACGCTGAGGCAGCGTCCCACATGCCACAACT--AGAGGA
CM009161.1:41796160-41796635     CTTGAATTTAAAAAGGCTAGCAGGGGGCTGGCCCCGTGGCCGAGTGGGTAAGTTCGCGCGCTCCGCTGCAGGCGGCCCAGTGTTTCGTCGGTTCGAATCCTGGGCGCGGACATGGCACTGCTCGTCAGACCACGCTGAGGCAGCGTCCCACATGCCACAACT--AGAGGA
CM009161.1:5817566-5817976       ATAGTTAATAAAATATTCACCAAGGGGCTGGCCCCGTGGCCGAGTGGTTAAGTTCGCGCGCTCCTCTGCAGGCGGCCCAGTGTTTCGTCGGTTCGAATCCTGGGCGCGGACATGGCACTGCTCGTCAGACCACGCTGAGGCAGCGTCCCACATGCCACAACT--AGAGGA
CM009162.1:18640565-18641029     GACCATAATGGAACTAAATTAGAGGGGCTGGCCCCGTGGCCGAGTGGTTAAGTTCGTGCGCTCCGCTGCAGGCGGCCCAGTGTTTCGTCGGTTCGAATCCTGGGCGCGGACATGGCACTGCTCGTCAGACCACGCTGAGGCAGCGTCCCACATGCCACAACT--AGAGGA
CM009162.1:72721499-72721909     TCCCTTTAAAATCAGGAATAGGAGGGGCTGGCCCCGTGGCCGAGTGGTTAAGTTCGCGCGCTCCGCTGCAGGCGGCCCAGTGTTTCGTTGGTTCGAATCCTGGGCGCGGACATGGCACTGCTCGTCAGACCACGCTGAGGCAGCGTCCCACATGCCACAACT--AGAGGA
CM009162.1:73705477-73705954     CGATTAAAAACCACACCAAACACGGGGCTGGCCCCGTGGCCGAGTGGTTAAGTTCGCGCGCTCCGCTGCAGGCGGCCCAGTGTTTCGTCGGTTCGAATCCTGGGCGCGGACATGGCACTGCTCGTCGGACCACGCTGAGGCAGCGTCCCACATGCCACAACT--AGAGGA
CM009162.1:89336081-89336491     TCATTAAAAACCAAAGCAAATCAGGGGCTGGCCCCGTGGCCGAGTGGTTAAGTTCGCGCGCTCCGCTGCAGGCGGCCCAGTGTTTCGTCGGTTCGAATCCTGGGCGCGGACATGGCACTGCTCGTCAGACCACGCTGAGGCAGCGTCCCACATGCCACAACT--AGAGGA
CM009163.1:15029100-15029510     AGTCTAAAAAAGTAGATATTTTCGGGGCTGGCCCCGTGGCCGAGTGGTTAAGTTCGCGCGCTCCGCTGCAGGCGGCCCAGTGTTTCGTCGGTTCGAATCCTGGGCGTGGACATGGCACTGCTCGTCAGACCACGCTGAGGCAGCGTCCCACATGCCACAACT--AGAGGA
CM009163.1:18249627-18250037     ACAGAGATAAGAATATCCATTGAGGGGCTGGCCCCGTGGCCGAGTGGTTAAGTTCGCGCGCTCCGCTGCAGGCGGCCCAGTGTTTCGTCGGTTCGAATCCTGGGCGCGGACATGGCACTGCTCGTCAGACCACGCTGAGGCAGCGTCCCACATGCCACAACT--AGAGGA
CM009163.1:27212901-27213344     TTTTTAAAAGGAAGAAAAAGAGGGGGGCTGGCCCCGTGGCCGAGTGGTTAAGTTCGCGCGCTCCGCTGCAGGCGGCCCAGTGTTTCGTCAGTTCGAATCCTGGGCGCGGACATGGCACTGCTCGTCAGACCACGCTGAGGCAGCGTCCCACATGCCACAACT--AGAGGA
CM009163.1:57006995-57007418     AGTCAAAGTAAAGAAAGATGGGAGGGGCTGGCCCCGTGGCCGAGTGGTTAAGTTCGCGCGCTCCGCTGCAGGCGGCCCAGTGTTTCGTCGGTTCGAATCCTGGGCGCGGACATGGCACTGCTCGTCAGACCACGCTGAGGCAGCGTCCCACATGCCACAACT--AGAGGA
CM009163.1:70208448-70208871     CAGCATAAAAATTACACCTTTTAGGGGCTGGCCCCGTGGCCGAGTGGTTAAGTTCGCGCGCTCCGCTGCAGGCGGCCCAGTGTTTCATCGGTTCGAATCCTGGGCGCGGACATGGCACTGCTCGTCAGACCACGCTGAGGCAGCGTCCCACATGCCACAACT--AGAGGA
CM009164.1:10017861-10018271     CAAAGAAGAAATACACATGACCAGGGGCTGGCCCCGTGGCCGAGTGGTTAAGTTCGCGTGCTCCGCTGCAGGCGGCCCAGTGTTTCGTCGGTTCGAATCCTGGGCGCGGACATGGCACTGCTCGTCAGACCACGCTGAGGCAGCGTCCCACATGCCACAACT--AGAGGA
CM009164.1:27451760-27452172     CTATGATAAAGAAAGCATAAATAGGGGCTGGCCCCGTGGCCGAGTGGTTAAGTTCGCGCGCTCCGCTGCAGGCGGCCCAGTGTTTCGTCGGTTCGAATCCTGGGCGCGGACATGGCACTGCTCGTCAGACCACGCTGAGGCAGCGTCCCACATGCCACAACT--AGAGGA
CM009164.1:44968956-44969398     TTCTTTAAAAGTTTAGCATTTTCGGGGCTGGCCCCGTGGCCGAGTGGTTAAGTTCGCGCGCTCCGCTGCAGGCGGCCCAGTGTTTCGTCGGTTCGAATCCTGGGCGCGGACATGGCACTGCTCGTCAGACCACGCTGAGGCAGCGTCCCACATGCCACAACT--AGAGGA
CM009164.1:67932570-67932984     TGGAGTATAAAATCTGAATTAGAGGGGCTGGCCCCGTGGCCGAGTGGTTAAGTTCGCGCGCTCCGCTGCAGGCGGCCCAGTGTTTCGTCAGTTCGAATCCTGGGCGCGGACATGGCACTGCTCGTCAGACCACGCTGAGGCAGCGTCCCACATGCCACAACTAGAGAGGA
CM009165.1:32277707-32278117     TTGATTAAAATTATTGAATTTCTGGGGCTGGCCCCGTGGCCGAGTGGTTAAGTTCGCGCGCTCCGCTGCAGGCGGCCCAGTGTTTCGTCGGTTCGAATCCTGGGCGCGGACATGGCACTGCTCGTCAGACCACGCTGAGGCAGCGTCCCACATGCCACAACT--AGAGGA
CM009165.1:42445660-42446083     GCTTTTAAAAAATCTATAACACTGGGGCTGGCCCCGTGGCCGAGTGGTTAAGTTCGCGCGCTCCGCTGCAGGCGGCCCAGTGTTTCGTCGGTTCGAATCCTGGGCGCGGACATGGCACTGCTCGTCAGACCACGCTGAGGCAGCGTCCCACATGCCGCAACT--AGAGGA
CM009165.1:60294557-60295018     TGTGTACAAAAAATACTGTGTCGGGGGCTGGCCCTGTGGCCGAGTGGTTAAGTTCGCGCGCTCCGCTGCAGGCGGCCCAGTGTTTCGTCGGTTCGAATCCTGGGCGCGGACATGGCACTGCTCGTCAGACCACGCTGAGGCAGCGTCCCACATGCCACAACT--AGAGGA
CM009165.1:61061220-61061630     ATGCATATAAAAGACAATCCCAAGGGGCTGGCCCCGTGGCCGAGTGGTTAAGTTCGCGCGCTCCGCTGCAGGCGGCCCAGTGTTTCGTTGGTTCGAATCCTGGGCGCGGACATGGCACTGCTCGTCAGACCACGCTGAGGCAGCGTCCCACATGCCACAACT--AGAGGA
CM009165.1:79392823-79393273     GTTATGTCAAAAACCCCAGGAGGGGGGCTGGCCCCGTGGCCGAGTGGTTAAGTTCGCGCGCTCCGCTGCAGGCGGCCCAGTGTTTCGTCGGTTCGAATCCTGGGCGCGGACATGGCACTGCTCGTCAGACCACGCTGAGGCAGCGTCCCACATGCTACAACT--AGAGGA
CM009165.1:79822517-79822940     ACACCATAAGAAAAGGGAAAAGTGGGGCTGGCCCCGTGGCCGAGTGGTTAAGTTCGCGCGCTCCGCTGCAGGCGGCCCAGTGTTTCGTCGGTTCGAATCCTGGGCGCGGACATGGCACTGCTCGTCAGACCACGCTGAGGCAGCGTCCCACATGCCACAACT--AGAGGA
CM009166.1:20148003-20148457     AGGGTCTTCAAAAAGGTAATTGAGGGGCTGGCCCCGTGGCCGAGTGGTTAAGTTCGCGCGCTCCGCTGCAGGCAGCCCAGTGTTTCGTCGGTTCGAATCCTGGGCGCGGACATGGCACTGCTCGTCAGACCACGCTGAGGCAGCGTCCCACATGCCACAACT--AGAGGA
CM009166.1:60671460-60671870     TCATTTTTAAAAACACTACCACTGGGGCTGGCCCCGTGGCCGAGTGGTTAAGTTCGCGCGCTCCGCTGCAGGCGGCCCAGTGTTTCGTCGGTTCGAATCCTGGGCGCGGACATGGCGCTGCTCGTCAGACCACGCTGAGGCAGCGTCCCACATGCCACAACT--AGAGGA
CM009166.1:917821-918231         TGTTGTAAGAAACACCAAGCTCTGGGGCTGGCCCCGTGGCCGAGTGGTTAAGTTCGCGCGCTCCGCTGCAGGCGGCCCAGTGTTTCGTCGGTTCGAATCCTGGGCGCGGACATGGCACTGCTCGTCAGACCACGCTGAGGCAGCGTCCCACATGCCACAACT--AGAGGA
CM009167.1:10379514-10379988     AGTTTACAATAAGTGAAAAAGTTGGGGCTGGCCCCGTGGCCGAGTGGTTAAGTTCACGCGCTCCGCTGCAGGCGGCCCAGTGTTTCGTCGGTTCGAATCCTGGGCGCGGACATGGCACTGCTCGTCAGACCACGCTGAGGCAGCGTCCCACATGCCACAACT--AGAGGA
CM009167.1:34882706-34883116     GTCTTAAAAACCACTGGCTCAGGGGGCTGGCCCCGTGGCCGAGTGGTTAAGTTCGCGCGCTCCGCTGCAGGCGGCCCAGTGTTTCGTCGGTTCGAATCCTGGGCGCGGACATGGCACTGCTCGTCAGACCACGCTGAGGCAGCGTCCCACATGCCACAACT--AGAGGA
```

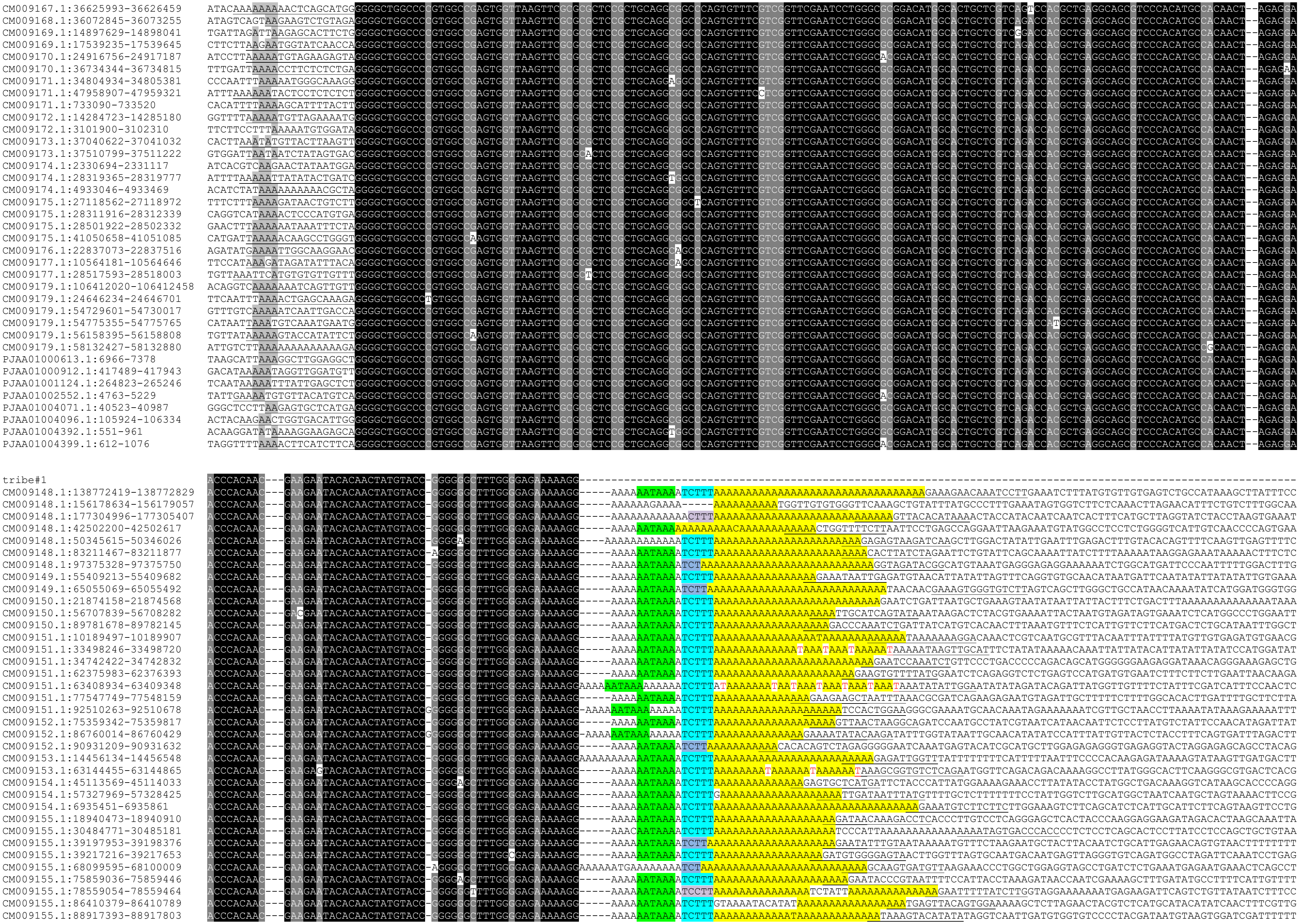
CM009167.1:36625993-36626459
CM009168.1:36072845-36073255
CM009169.1:14897629-14898041
CM009169.1:17539235-17539645
CM009170.1:24916756-24917187
CM009170.1:36734344-36734815
CM009171.1:34804934-34805381
CM009171.1:47958907-47959321
CM009171.1:733090-733520
CM009172.1:14284723-14285180
CM009172.1:3101900-3102310
CM009173.1:37040622-37041032
CM009173.1:37510799-37511222
CM009174.1:2330694-2331117
CM009174.1:28319365-28319777
CM009174.1:4933046-4933469
CM009175.1:27118562-27118972
CM009175.1:28311916-28312339
CM009175.1:28501922-28502332
CM009175.1:41050658-41051085
CM009176.1:22837073-22837516
CM009177.1:10564181-10564646
CM009177.1:28517593-28518003
CM009179.1:106412020-106412458
CM009179.1:24646234-24646701
CM009179.1:54729601-54730017
CM009179.1:54775355-54775765
CM009179.1:56158395-56158808
CM009179.1:58132427-58132880
PJAA01000613.1:6966-7378
PJAA01000912.1:417489-417943
PJAA01001124.1:264823-265246
PJAA01002552.1:4763-5229
PJAA01004071.1:40523-40987
PJAA01004096.1:105924-106334
PJAA01004392.1:551-961
PJAA01004399.1:612-1076
tribe#1
CM009148.1:138772419-138772829
CM009148.1:156178634-156179057
CM009148.1:177304996-177305407
CM009148.1:42502200-42502617
CM009148.1:50345615-50346026
CM009148.1:83211467-83211877
CM009148.1:97375328-97375750
CM009149.1:55409213-55409682
CM009149.1:65055069-65055492
CM009150.1:21874158-21874568
CM009150.1:56707859-56708282
CM009150.1:89781678-89782145
CM009151.1:10189497-10189907
CM009151.1:33498246-33498720
CM009151.1:34742422-34742832
CM009151.1:62375983-62376393
CM009151.1:63408934-63409348
CM009151.1:77547749-77548159
CM009151.1:92510263-92510678
CM009152.1:75359342-75359817
CM009152.1:86760014-86760429
CM009152.1:90931209-90931632
CM009153.1:14456134-14456548
CM009153.1:63144455-63144865
CM009154.1:45113569-45114033
CM009154.1:57327969-57328425
CM009154.1:6935451-6935861
CM009155.1:18940473-18940910
CM009155.1:30484771-30485181
CM009155.1:39197953-39198376
CM009155.1:39217216-39217653
CM009155.1:68099595-68100009
CM009155.1:75859036-75859446
CM009155.1:78559054-78559464
CM009155.1:86410379-86410789
CM009155.1:88917393-88917803

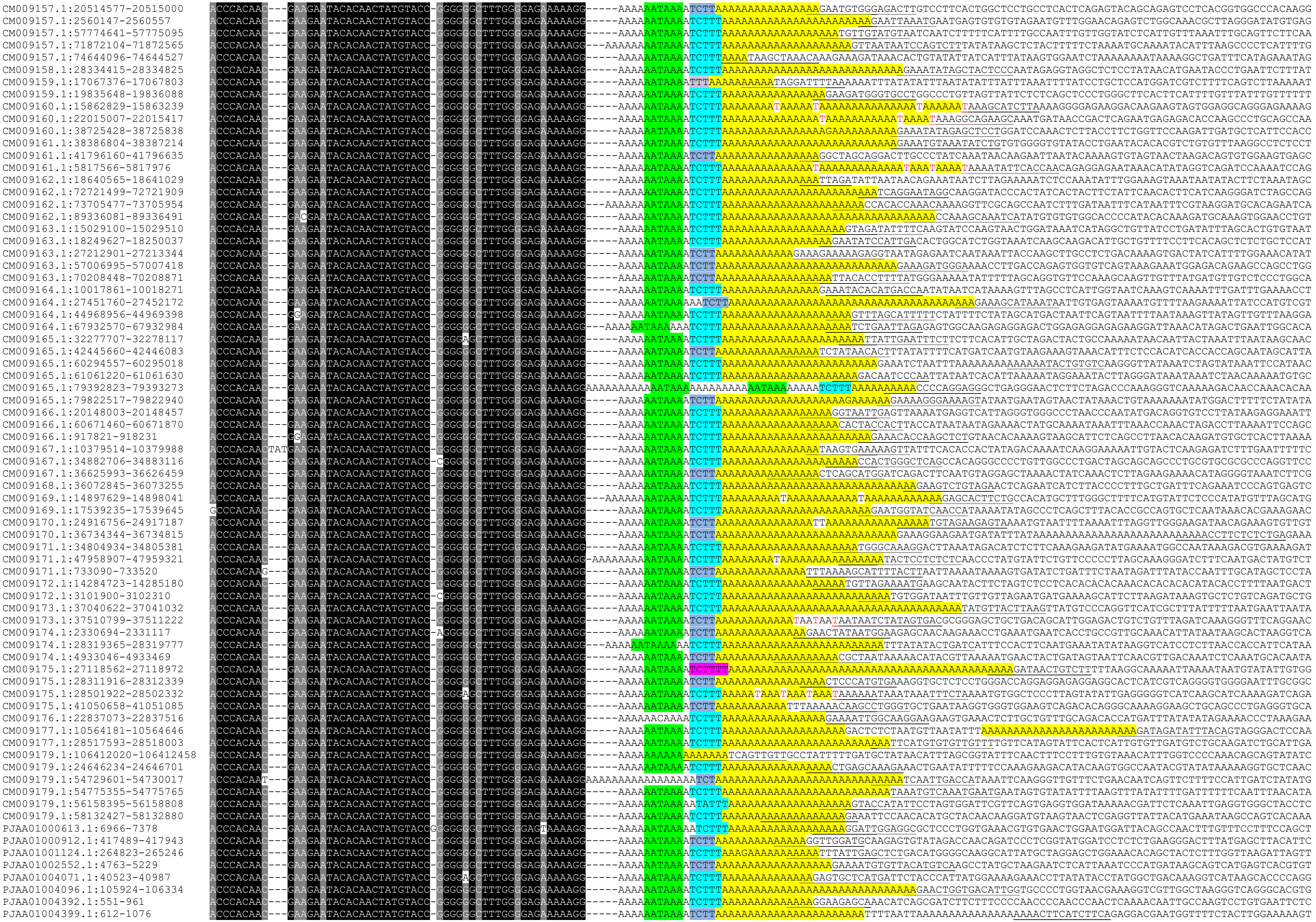

CM009157.1:20514577-20515000
CM009157.1:2560147-2560557
CM009157.1:57774641-57775095
CM009157.1:71872104-71872565
CM009157.1:74644096-74644527
CM009158.1:28334415-28334825
CM009159.1:17067376-17067803
CM009159.1:19835648-19836088
CM009160.1:15862829-15863239
CM009160.1:22015007-22015417
CM009160.1:38725428-38725838
CM009161.1:38386804-38387214
CM009161.1:41796160-41796635
CM009161.1:5817566-5817976
CM009162.1:18640565-18641029
CM009162.1:72721499-72721909
CM009162.1:73705477-73705954
CM009162.1:89336081-89336491
CM009163.1:15029100-15029510
CM009163.1:18249627-18250037
CM009163.1:27212901-27213344
CM009163.1:57006995-57007418
CM009163.1:70208448-70208871
CM009164.1:10017861-10018271
CM009164.1:27451760-27452172
CM009164.1:44968956-44969398
CM009164.1:67932570-67932984
CM009165.1:32277707-32278117
CM009165.1:42445660-42446083
CM009165.1:60294557-60295018
CM009165.1:61061220-61061630
CM009165.1:79392823-79393273
CM009165.1:79822517-79822940
CM009166.1:20148003-20148457
CM009166.1:60671460-60671870
CM009166.1:917821-918231
CM009167.1:10379514-10379988
CM009167.1:34882706-34883116
CM009167.1:36625993-36626459
CM009168.1:36072845-36073255
CM009169.1:14897629-14898041
CM009169.1:17539235-17539645
CM009170.1:24916756-24917187
CM009170.1:36734344-36734815
CM009171.1:34804934-34805381
CM009171.1:47958907-47959321
CM009171.1:733090-733520
CM009172.1:14284723-14285180
CM009172.1:3101900-3102310
CM009173.1:37040622-37041032
CM009173.1:37510799-37511222
CM009174.1:2330694-2331117
CM009174.1:28319365-28319777
CM009174.1:4933046-4933469
CM009175.1:27118562-27118972
CM009175.1:28311916-28312339
CM009175.1:28501922-28502332
CM009175.1:41050658-41051085
CM009176.1:22837073-22837516
CM009177.1:10564181-10564646
CM009177.1:28517593-28518003
CM009179.1:106412020-106412458
CM009179.1:24646234-24646701
CM009179.1:54729601-54730017
CM009179.1:54775355-54775765
CM009179.1:56158395-56158808
CM009179.1:58132427-58132880
PJAA01000613.1:6966-7378
PJAA01000912.1:417489-417943
PJAA01001124.1:264823-265246
PJAA01002552.1:4763-5229
PJAA01004071.1:40523-40987
PJAA01004096.1:105924-106334
PJAA01004392.1:551-961
PJAA01004399.1:612-1076

Figure S4. Nucleotide sequences of Ere_0TD copies of tribe #1, which are present in the domestic horse but absent from orthologous loci in the Przewalski horse. TSDs flanking SINE copies are underlined. Sequence signals are highlighted as follows: PAS, in green; TCTTT terminator, in cyan; TCTTTT terminator, in magenta; terminator rudiments (TCTT, TTT, TCT, etc.), in gray; and poly(A)-tails, in yellow. Red font highlights T residues in certain poly(A)-tails (apparently, such T residues in SINE tails arise through tandem amplification of the $TA_{3\text{-}5}$ site, which sometimes occurs at the TSD end [5]).
