# Supplementary material for: Ere, a Family of Short Interspersed Elements in the Genomes of Odd-Toed Ungulates (Perissodactyla)
Source: Animals (Basel). 2024 Jul 5;14(13):1982. doi: 10.3390/ani14131982 (PMC11240701; doi:10.3390/ani14131982)
Supplement: Supplementary file 1 [file animals-14-01982-s001.zip › Figures S1-S8 pdf/Figure S6.pdf]

A

TAGAAGGACCCACAACGAAGAATATACAACTATGTACTGGGGGGCTTTGGGGAGAAAAAGGAAAAAATAAAATCTTTTT

B

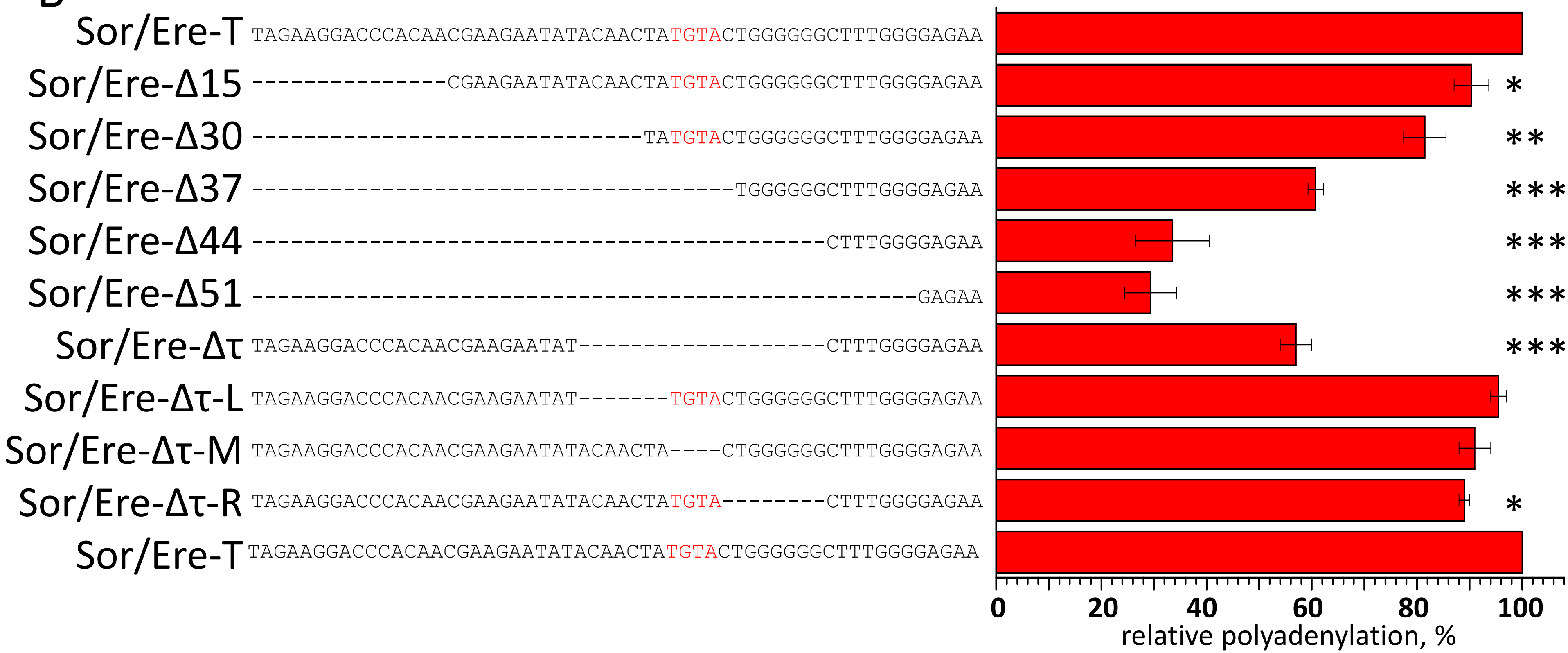

C

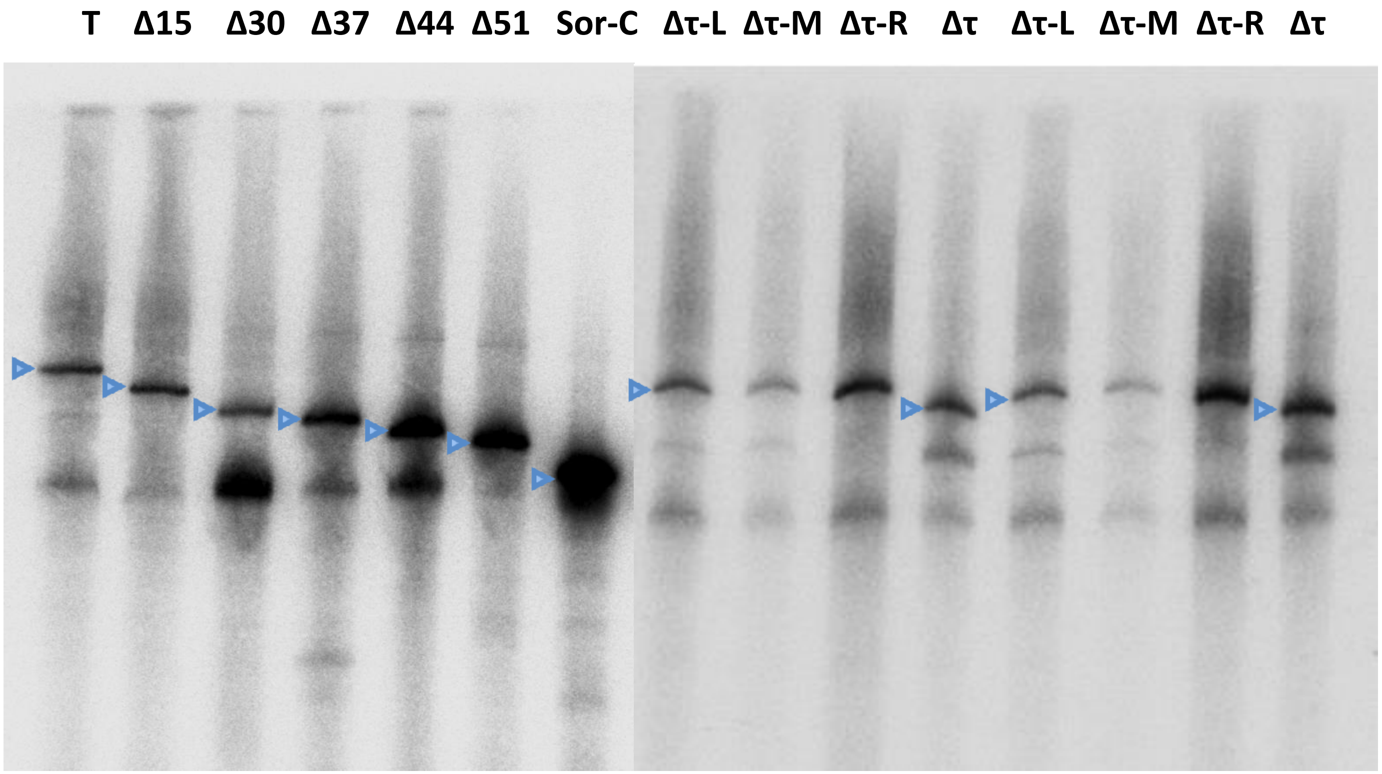

Figure S6. Effect of deletions in the putative τ region of Ere on polyadenylation of hybrid Sor/Ere transcripts.
A. The 3'-terminal region of Ere included in the Sor/Ere-T construct (see also Figure S1).
B. Diagram showing the relative polyadenylation level of transcripts of Sor/Ere constructs with deletions.
The polyadenylation level of the construct without deletions (Sor/Ere-T) was taken as 100% (shown in the top and bottom row of the diagram). The names of the constructs and the 3'-terminal sequence of Ere with deletions are shown on the left (deleted nucleotides are represented by dashes). The TGTA motif is indicated in red font.
(Error bars, SD, n = 3). ***$p \leq 0.001$; **$p \leq 0.01$; *$p \leq 0.05$. The p-value was determined by t-test.
C. Northern hybridization after transfection of HeLa cells with the above constructs. T is Sor/Ere-T and Sor-C is a Sor-based control construct (without the Ere region) whose transcripts are not polyadenylated. In each case, the smear above the top band (marked with an arrowhead) represents polyadenylated transcripts.
