# Supplementary material for: Ere, a Family of Short Interspersed Elements in the Genomes of Odd-Toed Ungulates (Perissodactyla)
Source: Animals (Basel). 2024 Jul 5;14(13):1982. doi: 10.3390/ani14131982 (PMC11240701; doi:10.3390/ani14131982)
Supplement: Supplementary file 1 [file animals-14-01982-s001.zip › Figures S1-S8 pdf/Figure S7.pdf]

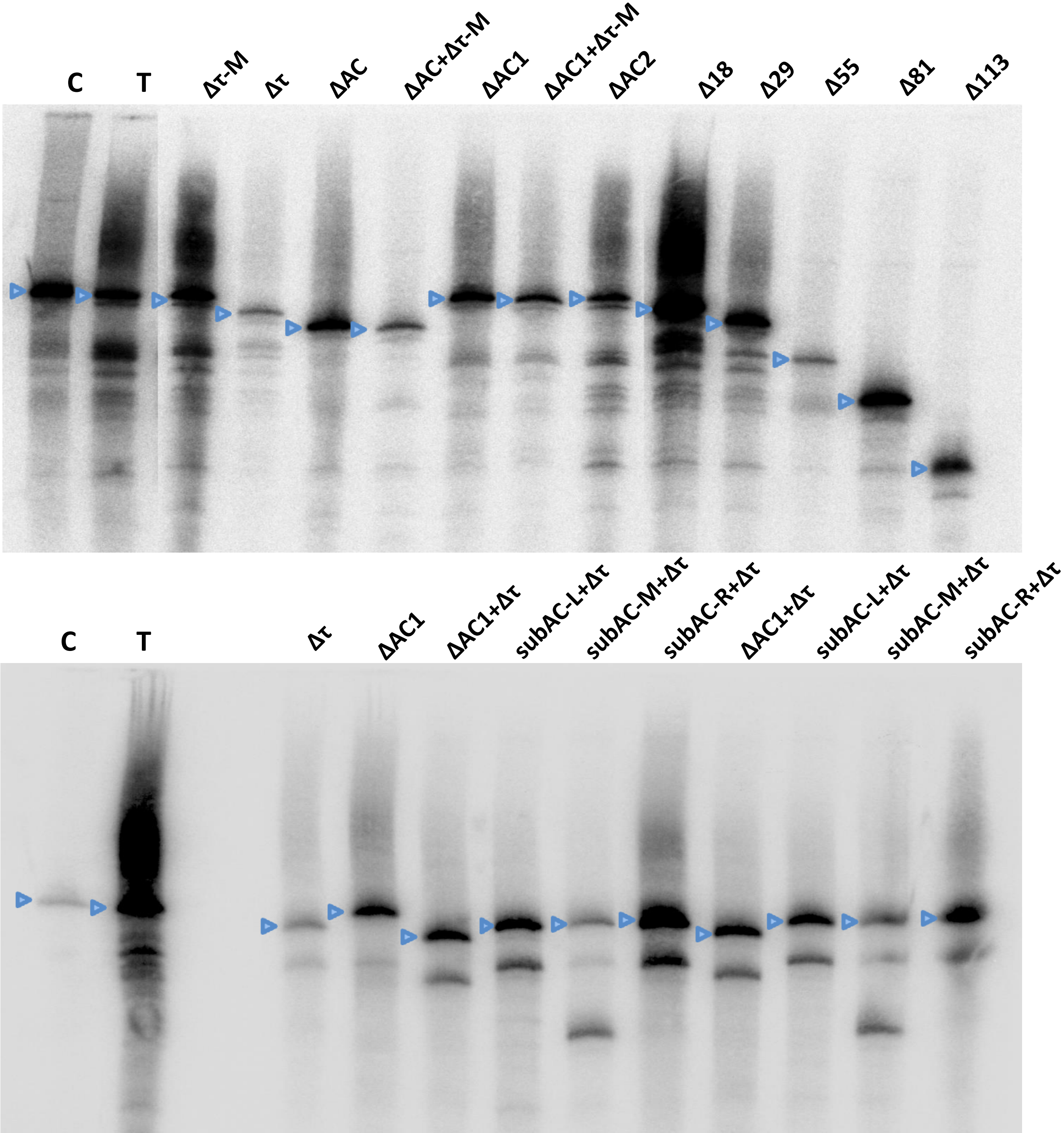

Figure S7. Selected Northern hybridization data obtained in HeLa transfection experiments, which shows the polyadenylation of transcripts of the constructs with the most effective mutations. The names of the construct are shown above the lanes. T and C correspond to Ere-T and Ere-C, respectively. The smear above the top band (marked with an arrowhead) represents polyadenylated transcripts.
