# Supplementary material for: Ere, a Family of Short Interspersed Elements in the Genomes of Odd-Toed Ungulates (Perissodactyla)
Source: Animals (Basel). 2024 Jul 5;14(13):1982. doi: 10.3390/ani14131982 (PMC11240701; doi:10.3390/ani14131982)
Supplement: Supplementary file 1 [file animals-14-01982-s001.zip › Figures S1-S8 pdf/Figure S8.pdf]

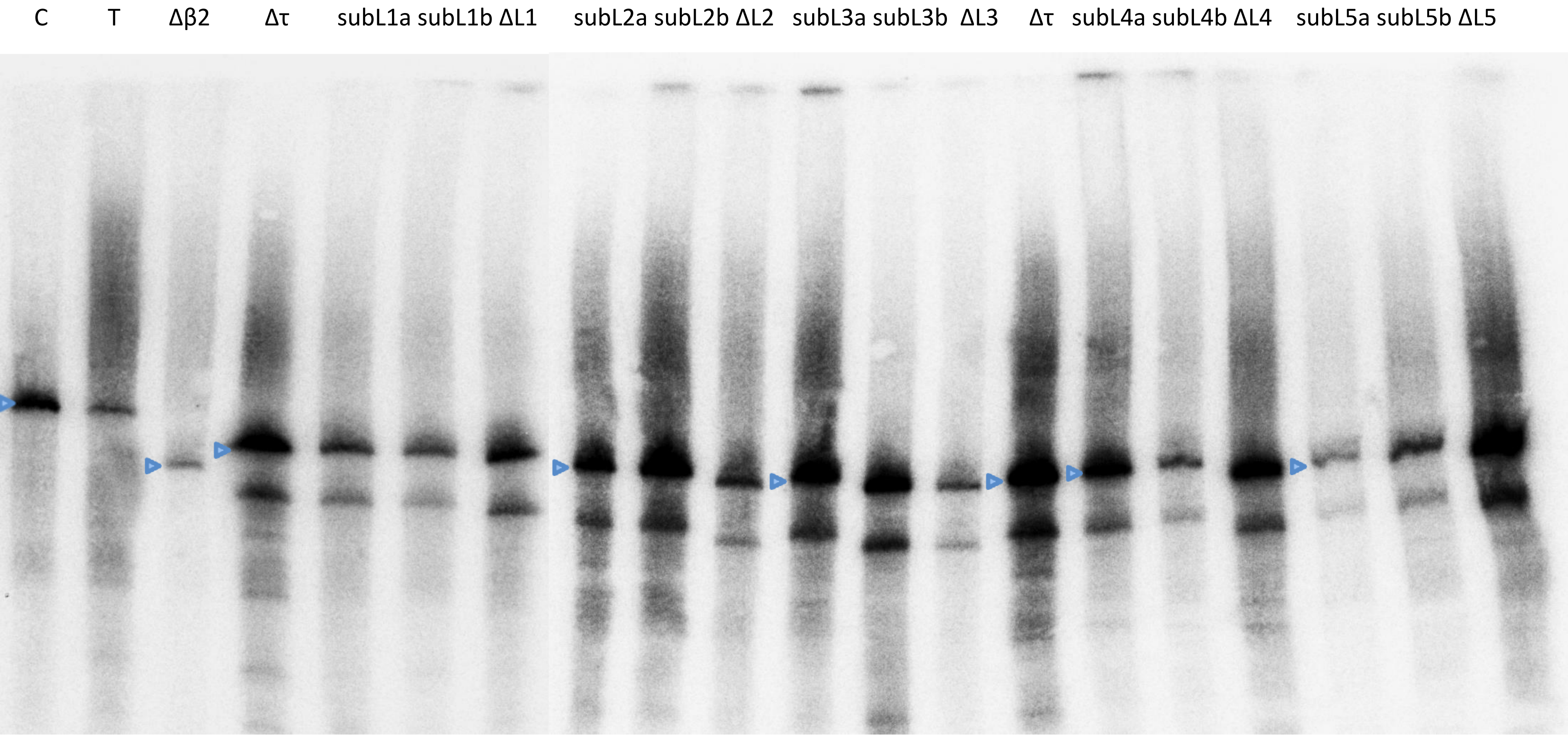


**Figure S8.** Northern hybridization analysis for HeLa cells transfected with Ere constructs carrying deletions or nucleotide substitutions upstream of the β region. The names of the constructs and the location of 6-nt deletions and 3-nt substitutions in the Ere sequence are provided in Figure 7. The smear above the top band (marked with the arrowhead) represents polyadenylated transcripts. The quantitative analysis of polyadenylation based on the results of this experiment is presented in Figure 7.
